# Microscale insight into the proton concentration during electrolytic reaction via an optical microfiber: potential for microcurrent monitoring by a dielectric probe

**DOI:** 10.1038/s41377-025-01770-9

**Published:** 2025-02-07

**Authors:** Yunyun Huang, Jiaxuan Liang, Haotian Wu, Pengwei Chen, Aoxiang Xiao, Bai-Ou Guan

**Affiliations:** 1 Guangdong Provincial Key Laboratory of Optical Fiber Sensing and Communications, Institute of Photonics Technology, Jinan University, Guangzhou, 511143 China; 2 College of Physics & Optoelectronic Engineering, Jinan University, Guangzhou, 510632 China

**Keywords:** Optical techniques, Fibre optics and optical communications

## Abstract

Local microcurrent monitoring is of great significance for biological and battery systems, yet it poses a formidable challenge. The current measurement techniques rely on electromagnetic materials which inevitably introduce interference to the system under examination. To address this issue, a promising approach based on a dielectric fiber-optic sensor is demonstrated. The microfiber is capable of detecting microcurrent through monitoring the localized proton concentration signal with a pH resolution of 0.0052 pH units. By sensing the refractive index variation surrounding the sensor induced by the interaction between local proton concentration changes and oxidizer-treated microfiber surface through the evanescent field, this sensing mechanism effectively avoids the interference of the electromagnetic material on the performance of the tested system. This sensor exhibits a limit of detection for microcurrent of 1 μA. The sensing region is a microfiber with a diameter of 8.8 μm. It can get invaluable information that cannot be obtained through conventional electrochemical methods. Examples include photocurrent attenuation in photogenerated carrier materials during illumination, electrical activation in nerve cells, and fluctuations in the efficiency of electrical energy generation during battery discharge. This approach provides a powerful complement to electrochemical methods for the elucidation of microscale reaction mechanisms. The information provided by the prepared dielectric fiber-optic sensor will shed more light on proton kinetics and electrochemical and electrobiological mechanisms, which may fill an important gap in the current bioelectricity and battery monitoring methods.

## Abstract (web summary)

A dielectric fiber-optic sensor is used to detect microcurrent through monitoring the localized proton concentration signal with a pH resolution of 0.0052 pH units.

## Graphical abstract

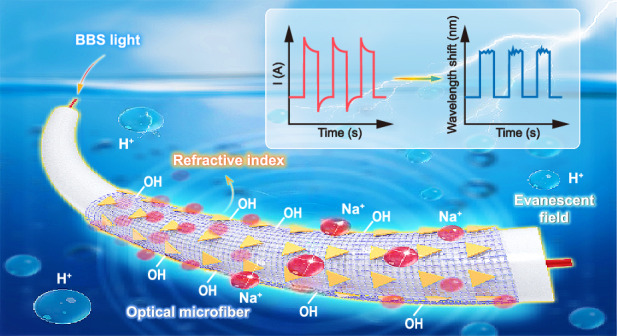

## Introduction

Many natural and engineered biochemical and electrochemical processes are regulated by the spatial distribution of the proton concentration in an aqueous solution, called the spatial distribution of the pH value^1–3^. This distribution directly controls the pathway, rate and product selectivity of electrolytic reactions^2^. Monitoring the spatial distribution of the proton concentration in an electrolyte is essential for understanding the kinetic processes, energy conversion efficiency, and system stability of electrochemical processes^2^. Additionally, this monitoring allows identification of corrosion hotspots and enables measures to prevent or mitigate material damage in terms of material corrosion to be taken^4^. In biomedical engineering, precise monitoring of proton concentrations facilitates the creation of microenvironments that closely resemble physiological conditions, thereby promoting cell growth or efficient drug release^5^. The change in the proton concentration at the electrodes in an electrolyte is even more intriguing, as it reflects the microcurrent of the system. Microcurrents play a critical role in the fields of battery materials, electrochemistry, and biological systems. Local microcurrent monitoring of neuronal populations facilitates the investigation of mapping from activity to dynamics at the cellular level, providing an optimal foundation for analyzing brain dynamics^6^. Additionally, in situ detection of local microcurrent is essential for monitoring battery charge and discharge processes and evaluating battery performance^7^. Since the generation of a microcurrent in a solution involves the directional transport of ions and the rearrangement of the spatial proton concentration in an electrolyte^8,9^, insight into the microcurrent of a system can be provided by monitoring the local proton concentration, especially the proton concentration at electrodes.

However, continuous onsite monitoring of local proton concentrations, particularly in biological or battery systems, at the microscopic level presents a great challenge. The challenge lies not only in the instrument resolution but also in the rapid diffusion of localized protons into the surrounding environment. The acquisition of transient surface localized proton concentration signals with high precision, rapidity, and high spatial resolution poses formidable difficulties. The majority of techniques for local proton concentration monitoring rely on microelectrode arrays^1,10,11^, which might introduce electromagnetic interference into the tested system. Electromagnetic interference may lead to diminished system performance during testing, data inaccuracies, operational malfunctions, and potential security vulnerabilities. If a dielectric probe can be used to detect the local proton concentration, it would effectively mitigate electromagnetic interference with the detection system. Notably, this capability would enable the seemingly impossible task of detecting microcurrents via a dielectric probe.

To realize local microcurrent monitoring without electromagnetic interference, in this work, we report the detection of the local proton concentration in an electrolyte via a dielectric optical microfiber sensor. The dielectric nature and chemical resistance of optical microfiber sensors, attributed to the use of silica glass materials^12–15^, ensure their lack of electromagnetic interference with the detection system. Owing to the presence of a submicron evanescent field, these sensors interact with substances on the surface at the submicron scale, thus perceiving information within this range^16,17^. However, in theory, the silica surfaces of optical microfibers are not responsive to changes in the proton concentration., i.e., the pH. We observed that after the surface of the optical microfibers is treated with strong oxidants, the response to the proton concentration can be effectively modulated through interactions with specific ions in an electrolyte, which changes the refractive index within a submicron range of the surface. Hence, the optical microfiber sensor can monitor the microcurrent of the measured system by sensing the proton concentration at a fixed site within the electrolyte. The sensor response to the microcurrent is synchronized with the variations in the proton concentration in the electrolyte accompanying the microcurrent variations. The dielectric sensor exhibited a limit of detection of 1 μA. This method enables identification of the current attenuation phenomenon of photogenerated carrier materials during illumination, the enhanced conductivity of nerve cells under electrical stimulation, and fluctuations in efficiency during battery power generation processes, which cannot be discerned via conventional techniques such as electrochemical methods. This approach provides a method for measuring local proton concentrations and microcurrents that minimizes interference with the system under investigation. It also provides a new method for in operando decoding of physical and chemical events at the microscale adjacent to the sensor.

## Results

### Monitoring setup and mechanism

**Fig. 1:**
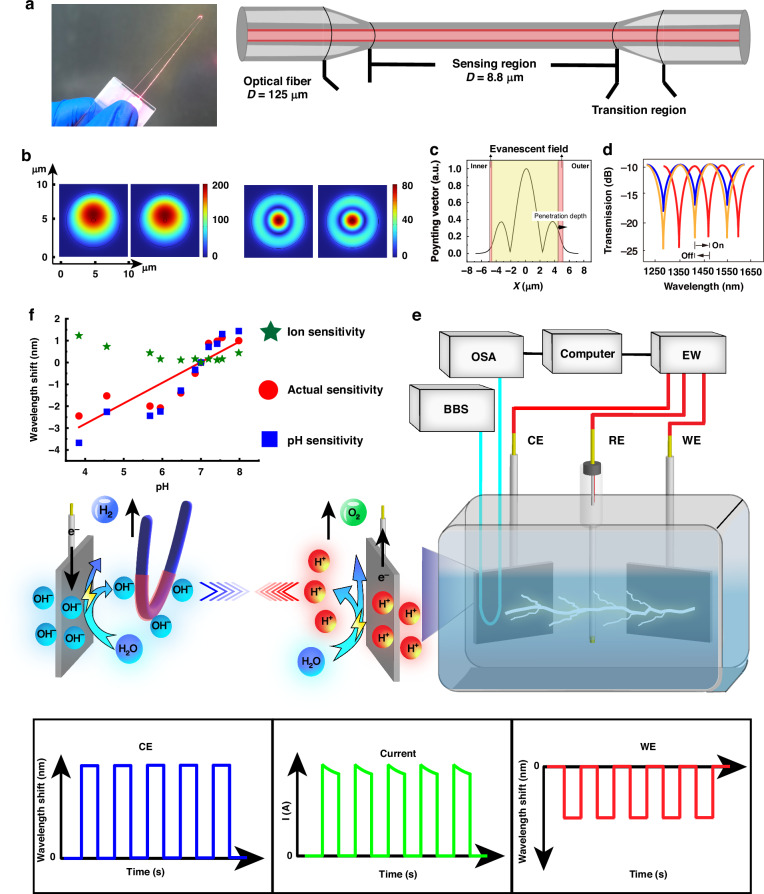
Concept of a dielectric fiber-optic sensor for sensing localized ion mobility. **a** Schematic diagram of the optical fiber sensing area. (Inset: photograph of the dielectric fiber-optic sensor). **b** Z-direction Poynting vectors of the bent silica microfiber at 1550 nm with a diameter of 8.8 μm calculated via numerical mode simulation software (HE_11_ and HE_12_ modes). For bending factor, the curvature radius (*r*) of the microfiber was 1.4 mm. **c** Transverse electric field amplitude distributions of the HE_12_ mode of the silica microfiber calculated via numerical mode simulation software. **d** Schematic diagram of the transmission spectrum utilized in localized proton concentration sensing. **e** Schematic diagram of the sensing setup. (BBS: broadband source; OSA: optical spectrum analyzer; EW: electrochemical workstation; CE: counter electrode; RE: reference electrode; WE: working electrode. The pulse signal in Fig. 1e is a schematic. During electrolysis, protons and hydroxyl ions were produced at the two electrodes and diffused towards the middle of the electrolyte). **f** pH sensitivity of the optical microfiber sensor without an interface. Ion interference: extra ions introduced during the pH regulation process. These ions caused changes in the refractive index of the solution, thereby affecting the measurement results of the sensor’s pH responses. Apparent pH sensitivity: the pH sensitivity of the sensor when sodium hydroxide or hydrochloric acid was added to the solution, which included refractive index interference caused by the extra ions. Actual pH sensitivity: the pH sensitivity subtracted the ‘ion interference’ from the ‘apparent pH sensitivity with ion interference’

To determine if a microcurrent could be monitored by the developed sensor, we first aimed to examine the sensing mechanism of the sensor via a numerical mode simulation. Considering factors such as sensitivity, portability, operability, cost, and fabrication difficulty, this work used a microfiber interferometer as a transducer. An optical microfiber with a uniform region of 8.8 μm in diameter and 622 μm in length and a transition region of 1.59 mm in length, as shown in Figs. 1a and S1, was employed in this work as a transducer. In theory, the smaller the diameter of the optical fiber is, the stronger the evanescent field, and the higher the sensitivity of the sensor. However, in practical applications, a fiber diameter that is too small results in excessive spectral loss and operation difficulty. For this trade-off, a microfiber with diameter of 8.8 μm was used in this work. In this work, the microfiber was bent in use. A single optical microfiber was used to complete all the detection applications. The optical microfiber was implanted in an electrolyte to monitor the local proton concentration. The microfiber was fabricated via a flame scanning method^18^. In this process, the microfiber was gradually elongated from a “normal” single-mode fiber to a taper structure connected to two single-mode silica optical fibers. This taper structure facilitated coupling of incident detection light and collection of the output signal (Fig. 1a), thus enabling the generation of both a fundamental mode (HE_11_ mode) and a high-order mode (HE_12_ mode)^19^, as calculated via the numerical mode simulation demonstrated in Fig. 1b. During the darwing process of the taper fiber, the cladding and core of the single-mode fiber fused to form the core of the microfiber. In addition, microfibers typically use low-index media such as air, vacuum, and water as their cladding^20^. The HE_11_ mode propagated within the core of the as-prepared microfiber, whereas the HE_12_ mode radially diffused out as an evanescent field (Fig. 1c) and interacted with the surrounding environment, such as the surrounding ions. Bending resulted in increased leakage of the evanescent field to the outer side of the bending region. Similarly, its penetration depth was also greater than that of the one without bending. Therefore, bending enhanced the sensor’s sensitivity. It has been reported that this sensitivity enhancement could be increased by up to 3 times around the refractive index of 1.333 Ref. ^21^. The bent microfiber with a cone diameter of 8.8 μm exhibited an evanescent field penetration depth of approximately 700 nm, enabling it to effectively sense environmental changes within this range. The superposition of the two modes, HE_11_ and HE_12_, gave rise to an interference pattern (Fig. 1d). Changes in the refractive index around the microfiber modulate the interferometric pattern shift, as determined by the following equation^22^

1 $$\frac{d\lambda }{d{n}_{{ext}}}=\lambda \cdot \frac{1}{\Gamma }\cdot \left(\frac{1}{\Delta {n}_{{eff}}}\cdot \frac{\partial \Delta {n}_{{eff}}}{\partial {n}_{{ext}}}\right)$$

In this equation, *n*_*ext*_ is the refractive index around the microfiber, $\varGamma =1-\frac{\lambda }{\Delta {n}_{{eff}}}\cdot \frac{d\Delta {n}_{{eff}}}{\partial {n}_{{ext}}}$ and *△n*_*eff*_ is the effective refractive index difference between the HE_11_ and HE_12_ modes. Consequently, an increase in the refractive index within the evanescent field surrounding the microfiber leads to a wavelength shift toward longer wavelengths (redshift), whereas a decrease in the refractive index results in a wavelength shift toward shorter wavelengths (blueshift). The resolution of optical microfibers for the refractive index can reach 10^-6^ RIU^20^. In this work, the spectral stability of the microfiber sensor without interface in aqueous solution is shown in Fig. S2. It indicates that the sensor exhibited a good stability in aqueous solution. Therefore, when we used an optical spectrum analyzer (OSA) with a resolution of 0.02 nm, the limit on the refractive index resolution of the sensor was determined primarily by the spectral resolution of the OSA.

Based on the above transducer, Fig. 1e shows the microcurrent excitation-monitoring system. The three-electrode system was driven by an electrochemical workstation. This electrochemical workstation was used to induce a microcurrent in the solution, resulting in a change in the spatial proton concentration distribution after electrification. Specifically, when an external voltage was applied, an oxidation reaction occurred at the anode (working electrode), and a reduction reaction occurred at the cathode (counter electrode). Water molecules were oxidized at the anode, releasing H^+^ (proton), while water molecules were reduced to produce hydrogen gas and hydroxide ions (OH^-^) at the cathode. With an external voltage applied, H^+^ and OH^-^ ions produced at the electrodes rapidly diffused, forming a concentration gradient in the electrolyte solution from electrodes to the middle of the solution. The concentration of H^+^ decreased from the anode to the middle of the solution, while the concentration of OH^-^ decreased from the cathode to the middle of the solution. The pH in the middle of the electrolyte remained unchanged. The dielectric optical microfiber was used to probe various positions in the electrolyte to monitor the localized proton concentration change. The proton concentration information measured when the sensor was attached to the surface of the working electrode or counter electrode can reflect the microcurrent information of the system. Thus, the sensor monitored the changes in the proton concentration at the working electrodes or counter electrode to indirectly monitor the system’s microcurrent. Before monitoring the microcurrents, we selected the electrode (working electrode or counter electrode) and used a microscope to measure the distance between the microfiber and the electrode precisely, and then fixed this distance before starting the microcurrent monitoring. In this work, when referring to microfiber at the electrode, the microfiber was 204 μm away from the electrode. This configuration also ensured strain-free operation of the sensor to eliminate the effect of the cross-sensitivity of the higher-order modes to the strain. A broadband source (BBS) with near-infrared light was employed to excite the spectrum of the microfiber sensor. From the perspectives of the absorption coefficient, the scattering effect, the influence of biological tissues and solutes, and the effect of light guidance by optical fiber, the near-infrared light provides a greater penetration depth and propagation length. An OSA was used to monitor the spectrum of the optical microfiber sensor. Both the optical and electrochemical signals were recorded and analyzed by a computer in real time.

The ability of the optical microfiber to detect variations in the proton concentration within an electrolyte is derived from its ability to detect changes in pH. We utilized sodium hydroxide and hydrochloric acid to adjust the pH of a sodium sulfate solution while employing a sodium chloride solution with an equivalent concentration to counteract any refractive index fluctuations resulting from the increase in the ion concentration. It is because during the evaluation pH responsiveness, the addition of sodium hydroxide or hydrochloric acid caused changes in the refractive index of the solution due to the introduction of extra ions. It thereby affected the wavelength shifts. This would cause interference from unwanted ions such as Cl^-^ and Na^+^ in the sensor response to pH. This is the ion interference. To eliminate the influence of the addition of excess ions on the refractive index, we used sodium chloride solutions of the same concentrations as the added hydrochloric acid or sodium hydroxide and recorded the sensor’s response to these concentrations of sodium chloride solution to obtain the wavelength shifts corresponding to the ion interference. We subtracted the wavelength shifts of the sensor to the ions at the same concentrations introduced (the ‘ion interference’) from the wavelength shifts responding to the addition of hydrochloric acid or sodium hydroxide (the ‘apparent pH sensitivity with ion interference’), and obtained the actual response of the sensor to pH (the ‘actual pH sensitivity’). As demonstrated in Fig. 1f, the silica optical microfiber exhibited a pH sensitivity of 0.942 nm per unit change in pH within the range of 4.0–8.0. This indicated that the ideal pH resolution of the silica optical microfiber was up to 0.021 pH units. (The ideal calculated pH resolution = spectrum resolution of the OSA/pH sensitivity^23^. This is the theoretical minimum detectable pH change. In practical applications, the pH resolution may also be affected by other factors such as background noise and temperature changes. As shown in Fig. S2, the background noise is small. Therefore, when measured at a fixed temperature, the pH resolution of the sensor can ideally be assumed to be 0.021 pH units.) Since the penetration depth of the evanescent field of the bent microfiber was 700 nm, theoretically, this is the surrounding area where this dielectric fiber can effectively detect. Therefore, the sensor with diameter of 8.8 μm is able to effectively distinguish the pH values of different spatial sites in the electrolyte with minor differences. Based on this pH sensing performance, the optical microfiber was applied to detect changes in the spatial proton concentration in the electrolyte when energized.

### Monitoring the local proton concentration at the working and counter electrodes

**Fig. 2:**
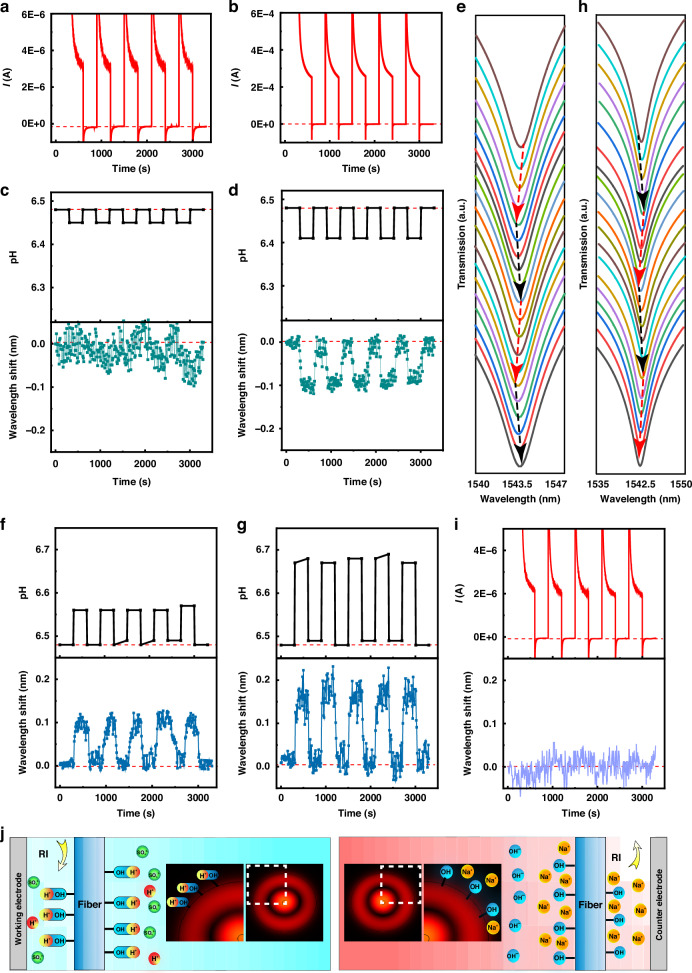
Optical responses of the sensor without an interface to various microcurrents in a solution. Electrochemical curves for microcurrents of (**a**) 3 μA and (**b**) 0.25 mA. **c**, **d** pH changes in the electrolyte at the working electrode with and without a voltage and the corresponding wavelength shifts recorded by the optical microfiber sensor at the working electrode for microcurrents. (**c**: microcurrent was 3 μA, (**d**): microcurrent was 0.25 mA). **e** Measured spectra corresponding to wavelength shifts in (**d**). **f**, **g** pH changes in the electrolyte at the counter electrode with and without a voltage and the corresponding wavelength shifts recorded by the optical microfiber sensor at the counter electrode for microcurrents. (f: microcurrent was 3 μA, g: microcurrent was 0.25 mA). **h** Measured spectra corresponding to wavelength shifts in (**g**). **i** Electrochemical curve and wavelength shifts recorded by the optical microfiber sensor at the counter electrode for a microcurrent of 2 μA. **j** Schematic diagram of the dielectric fiber mechanism for sensing proton concentration changes (inset: Schematic diagram of the Z-direction Poynting vector of the bent silica microfiber at 1550 nm in the proton change process (HE_12_ mode))

The synchronous optical responses of the sensor at the working electrode and counter electrode to microcurrents are illustrated in Fig. 2. Under a 3 μA current (Fig. 2a), the pH at the working electrode simultaneously decreased by 0.03 units. The pH promptly reverted to its initial level upon removal of the voltage (Fig. 2c). After the voltage was reapplied to the system, another pH fluctuation occurred. Upon removal of the voltage, the pH returned to its initial value, and this cycle was repeated. The sensor located at the working electrode was capable of responding to these pH fluctuations; however, the regularity was rather weak (Fig. 2c). When the amplitude of the periodic current stimulation was increased to 0.25 mA (Fig. 2b), there was a corresponding periodic decrease in the pH at the working electrode by 0.07 units (Fig. 2d). This pH alteration led to a sensor-recorded blueshift in transmission of 0.09 nm (Figs. 2d, e). Upon cessation of the current, both the pH and the spectrum returned to their initial values.

Figure 2f–h shows the results for the scenario in which the sensor was positioned on the counter electrode. As the current appeared and disappeared, the pH at the counter electrode experienced corresponding increases and recoveries (Fig. 2f, g). Specifically, when the current was 3 μA, the pH increased from 6.48 to 6.56, whereas when the current was 0.25 mA, it increased from 6.48 to 6.67. In response to changes in pH, the sensor transmission spectrum demonstrated synchronized redshifts and recoveries, resulting in wavelength shifts being recorded by the sensor in response to the microcurrent. A current of 3 μA resulted in a redshift of 0.1 nm, whereas a current of 0.25 mA led to an approximate redshift of 0.17 nm (Fig. 2f–h). Because the pH changes of the electrolyte caused by the same microcurrent at the working electrode were approximately half of those induced by the pH changes at the counter electrode and had opposite acid‒alkali alterations, the wavelength shifts recorded by the sensor at the working electrode were approximately half of those recorded at the counter electrode and displayed the opposite direction. Furthermore, the observed wavelength shifts were in line with the pH sensitivity of the sensor, as demonstrated in Fig. 1f. Hence, the response of the sensor to the microcurrent can be deduced to originate from its response to the pH variations induced by the microcurrent in the electrolyte.

Nevertheless, the silica surface of the optical microfiber is theoretically inert to fluctuations in pH. Hence, how does the sensor demonstrate sensitivity to changes in pH in this study? To clarify this issue, sodium hydroxide and hydrochloric acid were added to deionized water to regulate the solution pH and thereby verify whether the optical microfiber sensor truly possesses a linear response to pH variations within the range of 4.0 to 8.0. As shown in Fig. S3, the sensor failed to display a regular response to pH changes in deionized water. Additionally, the optical microfiber surface after sodium hydroxide treatment exhibited irregular responses to variations in pH. Therefore, the linear response of the sensor to changes in the pH of the electrolyte can be inferred to be related to the ions present in the electrolyte and the way the microfiber surface is treated.

Silica surfaces oxidized through plasma treatment have been reported to be abundant in oxygen-containing groups, thereby causing the silica surface to exhibit a regular response to pH changes^24,25^. The infrared spectroscopy results shown in Fig. S4, shows abundant oxygen-containing groups emerged^26,27^ on the silica surface of the optical microfiber after its treatment with piranha solution. Based on the reported results, these oxygen-containing groups play crucial roles in the pH sensitivity of silica in the electrolyte. Hence, we speculate the mechanism of pH sensitivity of the obtained sensor in sodium sulfate solution as shown in Fig. 2j. A uniform distribution of sodium ions and sulfate ions was formed around the optical microfiber featuring a negative oxygen-containing group on its surface when it was immersed in the electrolyte. When the electrolyte was energized, sodium ions migrated toward the counter electrode, whereas sulfate ions moved toward the working electrode. Simultaneously, protons (H^+^) were generated at the working electrode, whereas hydroxide ions were produced at the counter electrode. The hydrogen ions and hydroxide ions then diffused, resulting in concentration differences at various locations in the electrolyte. Consequently, the electrolyte near the working electrode became more acidic, whereas that near the counter electrode became more alkaline. When the microfiber was positioned at the working electrode, oxygen-containing groups on the silica surface attracted protons (H^+^) upon activation, creating an environment similar to that of water molecules. This resulted in a reduction in the refractive index of the electrolyte on the sensor surface, so a blueshift in the transmission could be recorded. When the microfiber was positioned at the counter electrode, the oxygen-containing groups on the silica surface repelled hydroxide ions and adsorbed sodium ions on the surface after the electrolyte was energized. This process increased the refractive index of the electrolyte on the sensor surface, resulting in a redshift in the transmission recorded by the sensor. In accordance with this principle, the sensor could monitor the pH changes in the electrolyte both before and after power activation, with a specific focus on the variation in the proton concentration. As proton concentration changes at a specific location within the electrolyte are correlated with the system microcurrent, the sensor could effectively monitor the microcurrent fluctuations. When the microcurrent was 2 μA (Fig. 2i), the corresponding pH change at the counter electrode was 0.02 units (Fig. S5), and the sensor might not have accurately recorded a regular wavelength shift (Fig. 2i). Thus, the current detection limit of the silica microfiber sensor was 3 μA at the working electrode, whereas it was 2 μA at the counter electrode.

### Interface functionalization

Given that the microcurrents in practical applications are typically extremely small, even reaching the level of nanocurrents, the resulting proton concentration change is correspondingly weak. Consequently, when employing fiber-optic sensors for monitoring spatial proton concentrations in biological or battery systems, enhancing their sensitivity is imperative. The utilization of high-index coatings has demonstrated the potential to increase the sensor sensitivity by extracting greater evanescent fields^28–30^. Additionally, the implementation of metal nanostructures has been proven to be effective in amplifying fiber evanescent fields through localized surface plasmon resonance effects, thereby further improving the sensor sensitivity^31–33^. In this work, we explore the enhancement of the fiber-optic sensor sensitivity through the utilization of these two phenomena.

**Fig. 3:**
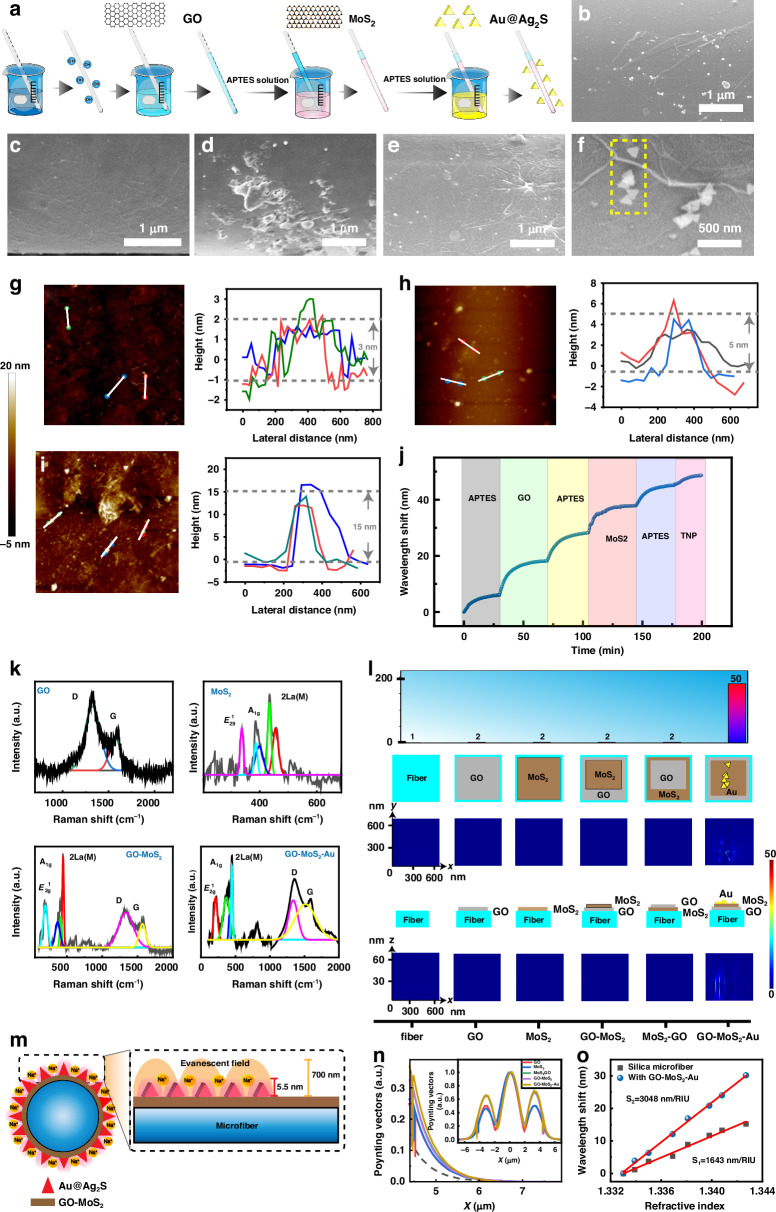
Interface functionalization. **a** Schematic diagram of the interface functionalization process. Scanning electron microscopy (SEM) images of microfiber surfaces with (**b**) MoS_2_, (**c**) GO, (**d**) MoS_2_-GO, **e** GO-MoS_2_, and (**f**) GO-MoS_2_-Au. (The position outlined by the dotted line in (**f**) was the structure model of Au@Ag_2_S in the FDTD simulation.) AFM height profiles of microfiber surfaces with (**g**) GO, (**h**) MoS_2_, and (**i**) GO-MoS_2_-Au. (Due to the limitations of AFM resolution, microfibers could not be used in (**g**, **h**, **i**). Instead, the same processed optical fibers with a diameter of 125 μm were used for measurement. The results can provide a reference for the thickness of the surface layer on the microfiber). **j** Wavelength shifts recorded by the sensor with the GO-MoS_2_-Au interface during the interface functionalization process. **k** Raman spectra of microfiber surfaces with various interfaces. **l** FDTD map and corresponding intensities of the optical microfiber surfaces with GO, MoS_2_, GO-MoS_2_, MoS_2_-GO, and GO-MoS_2_-Au interfaces. (Inset: the unit and structures of the simulated microfiber. The thickness of each layer was based on the height information obtained from the AFM results: GO was 3 nm; MoS_2_ was 5 nm; Au@Ag_2_S was 15 nm). **m** Schematic diagram of evanescent field enhancement induced by the plasmon effect. **n** Transverse electric field amplitude distributions of the HE_12_ mode of bent microfibers with various interfaces calculated via numerical mode simulation software. (Simulation model: the thickness of GO was 3 nm, refractive index was 3.5388 ref. ^51^; the thickness of MoS_2_ was 5 nm, refractive index was 3.9257 ref. ^52^. For Au@Ag_2_S, since its surface occupied area was only 6.33%, and the surrounding was water, its thickness was 15 nm, refractive index was 1.3639 [1.8205 ref. ^53^ ×6.33% + 1.3330_(refractive index of water)_×(1–6.33%)]. We idealized the coatings as being uniform.) **o** Bulk refractive index sensitivities of the microfiber with the GO-MoS_2_-Au interface and the fiber without an interface

As demonstrated in Fig. 3a, the sequential piranha solution and 3-aminopropyl-triethoxysilane (APTES) solution treatment enriched the silica surface of the optical fiber with amino groups^34^. Additionally, graphene oxide (GO) functionalized with carboxyl groups was adsorbed on the fiber surface through electrostatic interactions^35^. The APTES solution was subsequently employed for further treatment to increase the amino group enrichment on the fiber surface, followed by electrostatic attraction to modify MoS_2_ on the GO-coated fiber surface. Finally, the microfiber surface was further modified with APTES, and Au nanotriangles coated with Ag_2_S shells (Au@Ag_2_S) were electrostatically attached to the fiber surface, accomplishing the interface construction.

Scanning electron microscopy (SEM) images in Fig. 3b–f presents the surface morphology of the microfiber surfaces after the functionalization with MoS_2_, GO, GO-MoS_2_, MoS_2_-GO and GO-MoS_2_-Au interfaces, respectively. MoS_2_ and GO were uniformly distributed across the microfiber surface, forming wrinkles that facilitated subsequent material binding on the microfiber surface^36^. The aggregation observed in MoS_2_, which was thicker than GO, suggested that the use of GO as the first layer was more favorable for achieving interface regularity and a uniform distribution of the aforementioned materials. Au@Ag_2_S nanotriangles were dispersed on the MoS_2_ surface, forming a localized plasmon resonance layer.

The height profiles obtained from the atomic force microscopy (AFM) images in Fig. 3g–i and S6 revealed that the thickness of the GO on the fiber surface was approximately 3 nm (3-4 layers), whereas that of MoS_2_ was approximately 5 nm (few layers) and that of Au@Ag_2_S was 15 nm. The GO distributed on the MoS_2_ surface exhibited a greater tendency to agglomerate than the MoS_2_ distributed on the GO surface did (Fig. S6). The step-like redshift in the transmission spectrum (Fig. 3j, S7) also indicated a gradual increase in the fiber surface thickness and roughness during nanointerface construction. The presence of GO and MoS_2_ at the interface was indicated by the characteristic peaks in the Raman spectrum^37,38^, which were representative of these materials (Fig. 3k). As depicted in Fig. S8 and Table S1, the surface area of the microfiber with the GO-MoS_2_ interface was partially occupied by Au@Ag_2_S nanotriangles, accounting for 6.33 ± 1.82% of the surface area. These results indicate that the sensor surface was successfully functionalized with GO, MoS_2_, and Au@Ag_2_S.

After the interface construction was complete, the sensitivity enhancement of the interface was assessed. Given that the spectral extinction peak of Au@Ag_2_S was observed at 1450 nm^39^, which coincided with the frequency of the evanescent field, leveraging the localized surface plasmon resonance effect within this wavelength range is a plausible approach for enhancing the evanescent field. A finite-difference time-domain (FDTD) method was used to calculate the near-field intensity mapping of the microfiber surfaces with the interfaces over a near-infrared wavelength range of 1250–1650 nm (Fig. 3l). After the GO-MoS_2_-Au interface was assembled, the electric field was enhanced by a maximum of approximately 50-fold. For the majority of the edges of the Au@Ag_2_S nanoplates, their electric field enhancement was only about 20 times. (The structure model of Au@Ag_2_S nanotriangles in the FDTD simulation was based on the distribution of nanoplates outlined by the dashed line in Fig. 3f.) However, the Au@Ag_2_S nanotriangles without GO and MoS_2_ spacers only exhibited a modest 30-fold increase (the maximum enhancement position) in the localized electric field at the interface (Fig. S9). The localized electric field enhancement at the interface was attributed to the localized surface plasmon resonance effect of the Au@Ag_2_S nanotriangles^39^; the synergistic effect of the spacers effectively amplified this enhancement phenomenon^40,41^.

Figure 3m shows the interface enhancement mechanism. The evanescent field interacts with the material around the microfiber, and an increased energy in this region leads to a clearer and more sensitive perception of the changes in the surrounding environment by the microfiber^42^. The GO and MoS_2_ layers with a high refractive index pulled more of the HE_12_ mode out from the core to form an evanescent field, thereby augmenting the interaction between the evanescent field and its surrounding environment (Fig. 3n). The simulation models of Fig. 3n are described in Fig. S10. The presence of the Au@Ag_2_S nanotriangles within this evanescent field amplified the evanescent field of the fiber through the plasmon resonance effect. Moreover, the synergistic effect of the GO, MoS_2_, and Au@Ag_2_S nanotriangles further enhanced the plasmon enhancement effect, which enhanced the evanescent field of the microfiber to improve the sensitivity. Owing to the pronounced sensitivity enhancement effect at the interface, the bulk refractive index sensitivities of the microfibers with interfaces were markedly greater than that of the fiber without an interface (twofold increase, Fig. 3o and S11). Since the spectral resolution of the OSA was 0.02 nm, the refractive index resolution of the sensor without an interface was 1.21×10^-5^ RIU (0.02 nm/1643 nm/RIU), while the refractive index resolution of the sensor with the GO-MoS_2_-Au interface was 6.56×10^-6^ RIU (0.02 nm/3048 nm/RIU). The sensitizing effect of the GO-MoS_2_-Au interface on the microfiber was remarkably pronounced. The bulk refractive index enhancement of the GO-MoS_2_-Au interface was much lower than the maximum electric field enhancement of the local hot spots. This occurred because the maximum electric field position of the Au@Ag_2_S nanotriangles (the vertices of the triangles, enhanced by approximately 50 times) was only the local maximum electric field position. For the majority of the edges of the Au@Ag_2_S nanoplates, their electric field enhancement was only about 20 times. The surface area occupied by Au@Ag_2_S nanotriangles accounted for 6.33 ± 1.82%. This means that the position where the electromagnetic field was enhanced by 20 times occupied the surface area of the microfiber by approximately 6.33%. Notably, the thickness of the Au@Ag_2_S nanotriangles was only 15 nm, so the range of surface enhancement on the microfiber was only within a range of 15 nm (within 25 nm with the spacers) from the microfiber surface, not the entire evanescent field. Therefore, the enhancement of Au@Ag_2_S nanotriangles on the bulk refractive index sensitivity was far less than that of the local maximum electric field. But the enhancement of the sensitivity provided by the interfaces establishes a fundamental basis for monitoring the local proton concentration via microfibers with interfaces. In addition, it is worth noting that, for GO and MoS_2_ coatings, the thicker the coating was, the greater the evanescent field that leaked to the surface of the optical microfiber (Fig. S12), and thereby the higher the sensitivity was. However, for the solution modification method used in this work, an increase in the concentration of the modification solution would cause large-scale aggregation and nonuniformity, which would lead to a sharp increase in spectral loss (Fig. S13) and thereby affect the detection application. For the Au@Ag_2_S density, Au@Ag_2_S could bring about electric field enhancement hotspots on the optical microfiber surface. Undoubtedly, the more hotspots, the stronger the evanescent field, and the higher the sensitivity of the sensor. Nevertheless, the increase in the quantity of Au@Ag_2_S also significantly augmented the scattering effect, resulting in a great loss in the spectrum (Fig. S14) and thereby influencing the application of the sensor. Based on these trade-offs, in this work, while ensuring spectral quality, the thickness of the coatings and the density of Au@Ag_2_S were maximized as much as possible to enhance the sensitivity of the sensor. Consequently, the interface thickness and assembly density presented here were adopted.

### Monitoring of the local proton concentration at the working electrode by sensors with interfaces

**Fig. 4:**
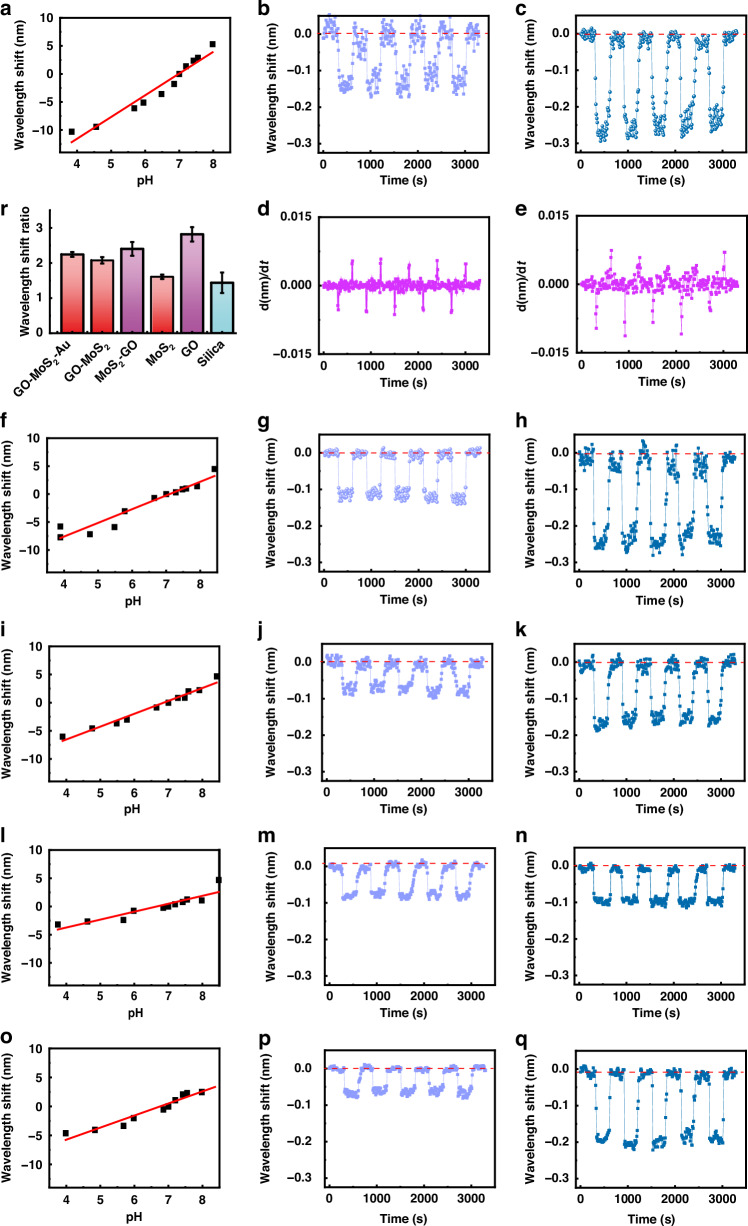
Optical responses of sensors with various interfaces under various microcurrents at the working electrode. Actual pH sensitivities in the electrolyte of sensors with (**a**) GO-MoS_2_-Au, (**f**) GO-MoS_2_, (**i**) MoS_2_-GO, **l** MoS_2_, and (**o**) GO interfaces. (Actual pH sensitivity: the pH sensitivity subtracted the ‘ion interference’ from the ‘apparent pH sensitivity with ion interference’.) Wavelength shifts of the sensors with (**b**, **c**) GO-MoS_2_-Au, (**g**, **h**) GO-MoS_2_, (**j**, **k**) MoS_2_-GO, (**m**, **n**) MoS_2_, and (**p**, **q**) GO interfaces. **b**, **g**, **j**, **m**, **p**: 3 μA; (**c**, **h**, **k**, **n**, **q**): 0.25 mA. Derivative of the wavelength shift with respect to time d(nm)/d*t* of the sensors for microcurrents of (**d**) 3 μA and (**e**) 0.25 mA. **r** Wavelength shift ratios when the microcurrent was 0.25 mA and 3 μA

Figures 4 and S15–S24 present the wavelength shifts recorded by the microfiber sensors with various interfaces at the working electrode under microcurrent stimulation. Figure 4a, f, i, l, o demonstrates the pH sensitivities of the microfibers with various interfaces in the electrolyte. The sensitivities were 3.872 nm/pH unit for the microfiber with the GO-MoS_2_-Au interface, 2.448 nm/pH unit for the microfiber with the GO-MoS_2_ interface, 2.285 nm/pH unit for the microfiber with the MoS_2_-GO interface, 2.098 nm/pH unit for the microfiber with the GO interface, and 1.404 nm/pH unit for the microfiber with the MoS_2_ interface. Thus, the pH resolution was up to 0.0052 pH units for the microfiber with the GO-MoS_2_-Au interface, up to 0.0082 pH units for the microfiber with the GO-MoS_2_ interface, up to 0.0088 pH units for the microfiber with the MoS_2_-GO interface, up to 0.0095 pH units for the microfiber with the GO interface, and up to 0.014 pH units for the microfiber with the MoS_2_ interface. Under a microcurrent, similar to the sensor without an interface, the sensors with various interfaces recorded rapid synchronous blueshifts at the working electrode. Upon cessation of the current, the spectrum promptly reverted back to its initial position through a redshift (Fig. 4b, c, g, h, j, k, m, n, p, q). As the current increased, the microfibers with various interfaces exhibited larger wavelength shifts. Under identical currents, the order of the wavelength shifts of the microfibers with different interfaces remained consistent with the order of their pH sensitivities, that is, sensor with GO-MoS_2_-Au > sensor with GO-MoS_2_ > sensor with MoS_2_-GO > sensor with GO > sensor with MoS_2_, in accordance with the order of the evanescent field enhancements depicted in Fig. 3n. It is noteable that for the MoS_2_-GO interface (Fig. 3d), it had a certain degree of aggregation due to the adjustment of the material assembly sequence (firstly MoS_2_ and then GO). Due to the influence of the morphology of MoS_2_, the surface was prone to have some aggregations. But, in the simulation of Poynting vector, we were unable to simulate such an aggregation effect, so we still idealized the use of a uniform coating model. In fact, these aggregations would slightly affect the sensor sensitivity. Therefore, although the Poynting vectors simulated for the GO-MoS_2_ and MoS_2_-GO interfaces were consistent, in reality, the sensitivity of MoS_2_-GO was slightly weaker. This interface was used as the control group.

The velocity peaks of the derivative of the wavelength shift with respect to time (Fig. 4d, e), which represent the highest rate of change in the wavelength shift during the process of the pulse current appearing and disappearing, were synchronized with the points in time when the pH change obtained by the pH meter reached its maximum and minimum values. This result indicated that the variation in the concentration of protons within the electrolyte was nearly concurrent with the emergence of the current (within a few tens of seconds). Thus, the optical signals captured by the sensor not only reflect the current in the electrolyte but also indicate the rate of change in the proton concentration in the solution, which is closely associated with variations in the electrolyte current. Moreover, the sensors with interfaces exhibited more pronounced differences in their optical responses to microcurrents of different intensities (3 μA and 0.25 mA) than did the sensor without an interface. The formers presented larger optical response ratios between high and low currents, as shown in Fig. 4r. This was also associated with their higher pH resolutions. Therefore, the enhanced intensity of the evanescent field increases the sensitivity of the sensor in detecting the local proton concentration and distinguishing subtle variations in microcurrents.

### Monitoring of the local proton concentration at the counter electrode by sensors with interfaces

**Fig. 5:**
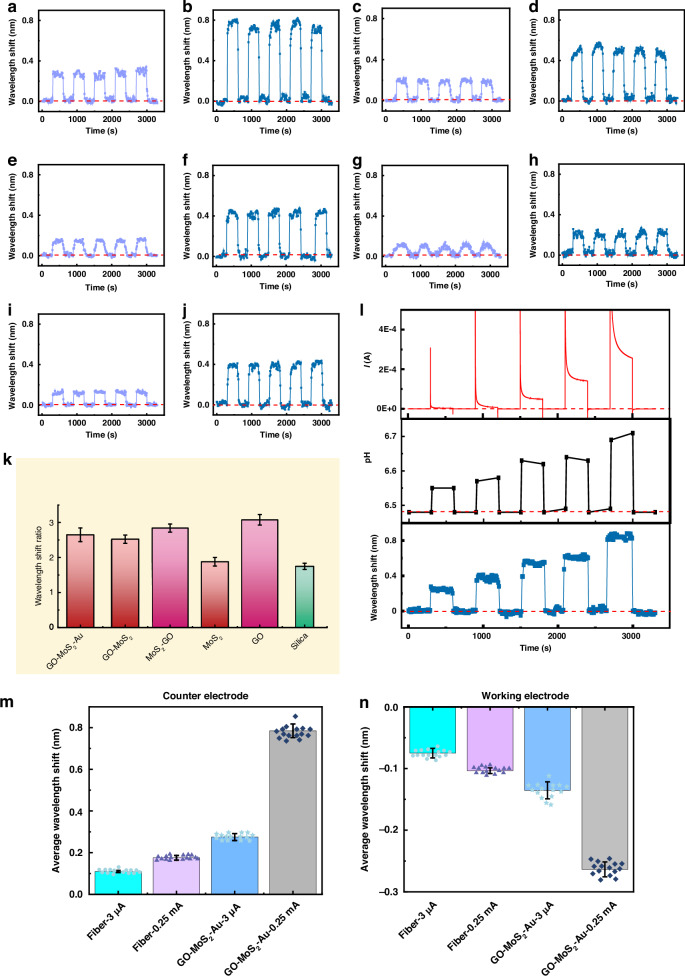
Optical responses of the sensors with various interfaces to various microcurrents at the counter electrode. Wavelength shifts of the sensors with (**a**, **b**) GO-MoS_2_-Au, (**c**, **d**) GO-MoS_2_, (**e**, **f**) MoS_2_-GO, (**g**, **h**) MoS_2_, and (**i**, **j**) GO interfaces. **k** Wavelength shift ratios when the microcurrent was 0.25 mA and 3 μA. (**a**, **c**, **e**, **g**, **i:** 3 μA; **b**, **d**, **f**, **h**, **j:** 0.25 mA). **l** Electrochemical curve, pH values and wavelength shifts of the sensor for increasing microcurrent from 3 μA to 0.25 mA. Average results of multiple measurements recorded by the sensor without interface and sensor with GO-MoS_2_-Au interface at (**m**) the counter electrode and (**n**) the working electrode, respectively. (*mean* ± SD, *n* = 15)

In addition, the local proton concentration at the counter electrode was also monitored by the sensors with interfaces to determine the difference in their response at the working and counter electrodes. Figures 5 and S25–S34 present the wavelength shifts recorded by the microfiber sensors with various interfaces at the counter electrode under microcurrent stimulation. As currents of 3 μA and 0.25 mA flowed through the system, the sensors with various interfaces recorded increased wavelength shifts as the current intensity increased (Fig. 5a–j). When the same current was applied to the electrolyte, the sequence of wavelength shifts detected by the sensors with various interfaces was as follows: sensor with GO-MoS_2_-Au > sensor with GO-MoS_2_ > sensor with MoS_2_-GO > sensor with GO > sensor with MoS_2_, in accordance with the order of the evanescent field enhancements depicted in Fig. 3n and the results displayed in Fig. 4. Interestingly, the wavelength shifts recorded by the sensors at the working electrode presented an opposite trend to those recorded at the counter electrode. Moreover, the amplitude of the wavelength shift was approximately twice that observed at the working electrode, which was in line with the results for the sensor without an interface and ultimately conformed to the magnitude of the pH change at the two electrodes under the same current. Consistent with the results acquired at the working electrode, the sensors with interfaces demonstrated higher optical response ratios between high and low currents (Fig. 5k). This result indicated that the intensified evanescent field enabled greater sensitivity of the sensor in detecting the local proton concentration and discriminating subtle variations in microcurrents. With the gradual increase in the system current (Fig. 5l), the pH measured with a pH meter at the counter electrode gradually increased, and the sensor with the GO-MoS_2_-Au interface recorded a corresponding gradual increase in redshift (Figs. 5l and S35). This result further demonstrated the ability of the optical microfiber sensor to monitor the microcurrent by tracking changes in the proton concentration at specific sites in the electrolyte. As shown in Fig. S36, when the microcurrent decreased to 2 μA, the sensor with the GO-MoS_2_-Au interface demonstrated a discernible and stable optical response. When the current further decreased to 1 μA, the sensor with the GO-MoS_2_-Au interface exhibited an irregular optical response (Fig. S37). Therefore, the GO-MoS_2_-Au interface enhanced the microcurrent detection limit of the optical microfiber. In addition, Fig. 5m, n shows the average results of multiple measurements (*mean* ± SD, *n* = 15). The small error bars reveal that the sensor has a good stability.

### Optical responses at various spatial locations

**Fig. 6:**
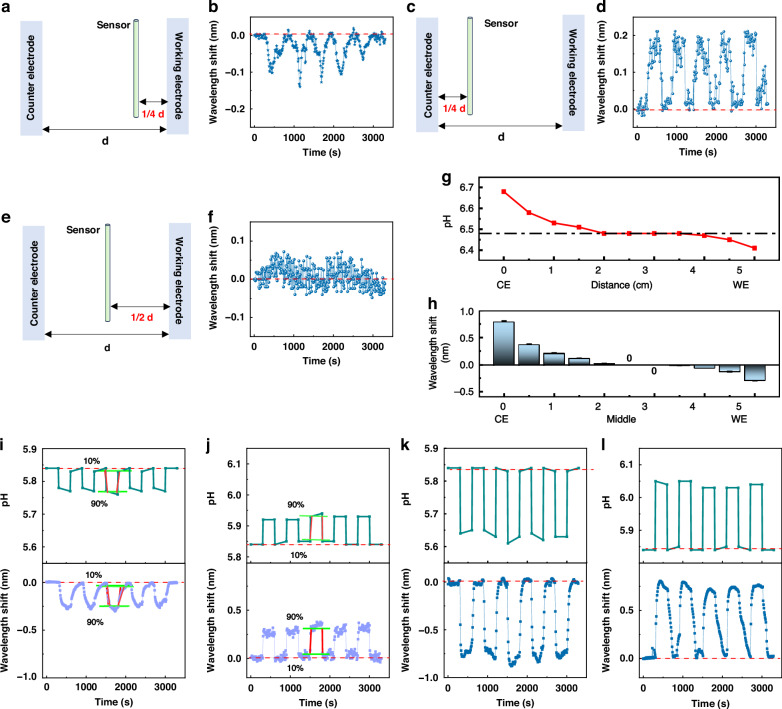
Optical responses of the sensors with and without the GO-MoS_2_-Au interface at various positions in the electrolyte. **a** Schematic diagram and (**b**) recorded wavelength shifts of the sensor with the GO-MoS_2_-Au interface at a position 1/4 of the distance from the working electrode when the current was 0.25 mA. **c** Schematic diagram and (**d**) recorded wavelength shifts of the sensor with the GO-MoS_2_-Au interface at a position 1/4 of the distance from the counter electrode when the current was 0.25 mA. **e** Schematic diagram and (**f**) recorded wavelength shifts of the sensor with the GO-MoS_2_-Au interface at the middle position between the working electrode and the counter electrode when the current was 0.25 mA. **g** pH changes and (**h**) wavelength shifts at various positions in the electrolyte when the current was 0.25 mA. pH changes and recorded wavelength shifts at the working electrode when the electrolyte was potassium chloride. (**i**: current was 3 μA; **k**: current was 0.25 mA). pH changes and recorded wavelength shifts at the counter electrode when the electrolyte was potassium chloride (**j**: current was 3 μA; **l**: current was 0.25 mA)

Since the optical microfiber with the GO-MoS_2_-Au interface exhibited the best optical response, it was studied as the prepared sensor in the following work. When the sensor with the GO-MoS_2_-Au interface was positioned at 1/4 of the distance between the working and counter electrodes (closer to the working electrode), synchronous wavelength shifts of approximately 0.1 nm could be observed in response to current changes (Fig. 6a, b; the current was 0.25 mA). When the prepared sensor was positioned at 1/4 of the distance between the working and counter electrodes (closer to the counter electrode), synchronous wavelength shifts of approximately 0.2 nm could be observed in response to current changes (Fig. 6c, d). At these two symmetric positions, the extent of the wavelength shift near the working electrode remained half of that near the counter electrode and in the opposite direction. When the sensor was positioned in the middle between the working and counter electrodes (Fig. 6e), no obvious wavelength shift was detected (Fig. 6f). We then monitored the variation in the proton concentration (pH) changes at diverse locations within the electrolyte when the current was 0.25 mA (Fig. 6g). For two locations about a quarter of the way from the two electrodes, when the electrolyte was sodium sulfate, the pH change in the vicinity of the working electrode was approximately half that in the vicinity of the counter electrode, and the pH change directions at the two positions were opposite. The pH change in the middle between the two electrodes was 0. Correspondingly, in line with the pH variation at each location, the sensor recorded a wavelength shift at that location with the exact same magnitude and direction as the pH changed (Fig. 6h). This finding substantiates that the fabricated dielectric sensor can achieve microscale perception of the proton concentration during an electrolytic reaction.

Furthermore, when the electrolyte was replaced with a potassium chloride solution (the electrolyte exhibited a balanced ratio of positive and negative ions, maintaining a 1:1 proportion), the pH variations induced by identical currents at the working electrode and counter electrode were identical, yet the directions were opposite (Fig. 6i-l). Accordingly, the wavelength shifts recorded by the sensor at the working electrode and counter electrode remained consistent but in opposite directions and were also consistent with those recorded at the counter electrode in the sodium sulfate solution. The disparity in the pH alterations at identical locations within the two electrolytes stems from the dissimilarity in the quantity of protons generated between the two electrodes resulted from the variance in the charge of the anions. This finding also indicates that the prepared sensor can monitor variations in the proton concentration at different positions within an energized electrolyte.

### Application demonstration of the microfiber sensor

The capacity to monitor changes in the local proton concentration endows the microfiber sensor with the capacity to monitor microcurrents. As shown in Fig. 6i, j, we evaluated the response time of the sensor. The rise (fall) process corresponded to the presence (removal) of applied voltage. We found that the rise/fall time was 55 s/48 s at the working electrode (Fig. 6i), and 20 s/5 s at the counter electrode (Fig. 6j) following the 10%-90% rule^43^. Such a response speed for a current sensor was indeed not very fast, but this response delay was not the delay of the fiber. It was mainly due to the fact that the microcurrent monitoring was derived from the measurement of proton concentration changes (pH changes) in the electrolyte, which were caused by the diffusion of protons and hydroxyl groups in the electrolyte when the current was applied. This process also took 55 s (rise time)/48 s (fall time) at the working electrode, and 20 s (rise time)/8 s (fall time) at the counter electrode. This led to the sensor’s response time being measured in seconds. Therefore, the sensor’s response to pH changes occurred almost simultaneously, whereas its response to current was somewhat delayed because of its detection mechanism. Since the response time of the sensor at the counter electrode was significantly shorter than that at the working electrode, we placed the sensor at the counter electrode for monitoring in chemical and biological applications. Despite not being very fast in response time, as a dielectric probe for microcurrent monitoring, our sensor is still capable of monitoring many biological or chemical processes with microcurrents that do not require extremely fast responses, such as the identification of photoinduced carrier decay, the monitoring of hydrogen‒oxygen battery power generation, and the monitoring of the polarization process of nerve cells. In the following work, the sensor was used in these applications. Because the signal acquired by the sensor at the counter electrode is more distinct, the sensor was uniformly positioned at the counter electrode for monitoring real samples. The microfiber was positioned at a distance of 204 μm from the counter electrode.

#### Identification of photoinduced carrier decay

**Fig. 7:**
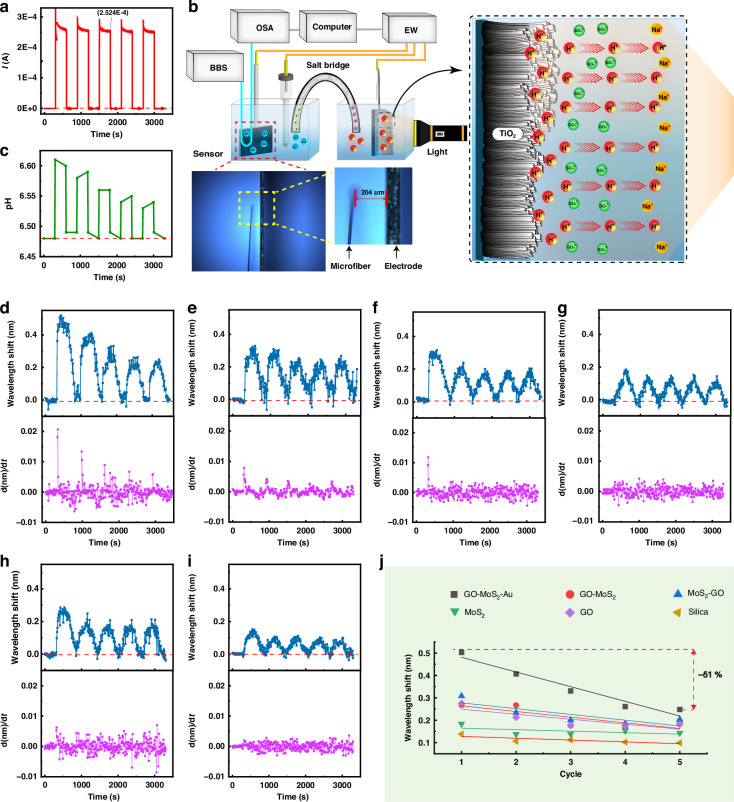
Optical responses of the sensors with various interfaces during monitoring of the photogenerated current of TiO_2_. **a** Photogenerated current of TiO_2_. **b** Schematic diagram of optical fiber monitoring of the photogenerated current of TiO_2_. Inset: microscopic photo of the microfiber position. The microfiber was positioned at a distance of 204 μm from the counter electrode. (BBS broadband source, OSA optical spectrum analyzer, EW electrochemical workstation, CE counter electrode, RE reference electrode, WE working electrode). **c** pH changes at the counter electrode during the photocurrent generation process. Wavelength shifts and derivative of the wavelength shift with respect to time d(nm)/d*t* of the sensors with (**d**) GO-MoS_2_-Au, (**e**) GO-MoS_2_, (**f**) MoS_2_-GO, (**g**) MoS_2_, and (**h**) GO interfaces and (**i**) without an interface. **j** Wavelength shift decay with increasing number of illumination cycles of TiO_2_

Monitoring the photogenerated microcurrent in photocurrent materials is an essential approach for evaluating their performance^44^. Here, the prepared dielectric sensor was employed for real-time monitoring of the photogenerated microcurrent in a TiO_2_ film. Under illumination, the TiO_2_ film exhibited a photocurrent of 0.25 mA, which could be consistently generated in the dark‒light alternating mode (Fig. 7a, b). Notably, when an electrochemical workstation was employed for monitoring the generation and disappearance of microcurrents, no discernible charge carrier attenuation phenomenon was observed. Nevertheless, with increasing number of light‒dark cycles, the increase in the pH at the electrode gradually diminished (Fig. 7c), which was not reflected in the current value monitored by the electrochemical workstation.

Dielectric fiber‒optic sensors with and without interfaces were placed in this system to monitor the photocurrent generated by the TiO_2_ film synchronously, as shown in Fig. 7d–i. The optical responses of all the fiber-optic sensors, regardless of whether they had interfaces, redshifted in the presence of microcurrents and blueshifted in their absence. Under the same microcurrent, as the sensitivity increased, the wavelength shift became more pronounced. It is worth noting that the optical responses recorded by the sensor without an interface (Fig. 7i) consistently agreed with the current values recorded by the electrochemical workstation. With an increase in the number of illumination cycles, no discernible attenuation phenomenon of the photocurrent or spectral response was observed. However, the optical responses recorded by the sensors with interfaces exhibited evident attenuation of the wavelength shifts (Fig. 7d–h). A stronger sensitization effect corresponded to a more pronounced microcurrent attenuation (Fig. 7d). The optical response attenuation caused by the photogenerated microcurrents under multiple irradiation cycles is illustrated in Fig. 7j. The results indicated that the sensor without an interface did not record any significant wavelength shift attenuation, whereas the sensor with the GO-MoS_2_-Au interface recorded a wavelength shift attenuation of over 50% during the fifth irradiation cycle, indicating microcurrent (carrier) attenuation. This result was in line with the variation in pH at this location. TiO_2_ photoelectrochemical materials have been reported to exhibit current attenuation after multiple irradiation cycles^45,46^. Consequently, the sensor with the GO-MoS_2_-Au interface could clearly detect this attenuation, whereas the sensor without an interface and the electrochemical workstation failed to do so. This finding aligns with the observations presented in Figs. 4 and 5, wherein the sensor with the GO-MoS_2_-Au interface effectively captured optical discrepancies between the different microcurrents, even when these differences were minute. Thus, the optical microfiber with GO-MoS_2_-Au interface provided the evidence of photocurrent attenuation in the carrier reaction, facilitating further investigation into materials that generate photocurrents.

#### Monitoring of the conductivity of neuronal cell populations

**Fig. 8:**
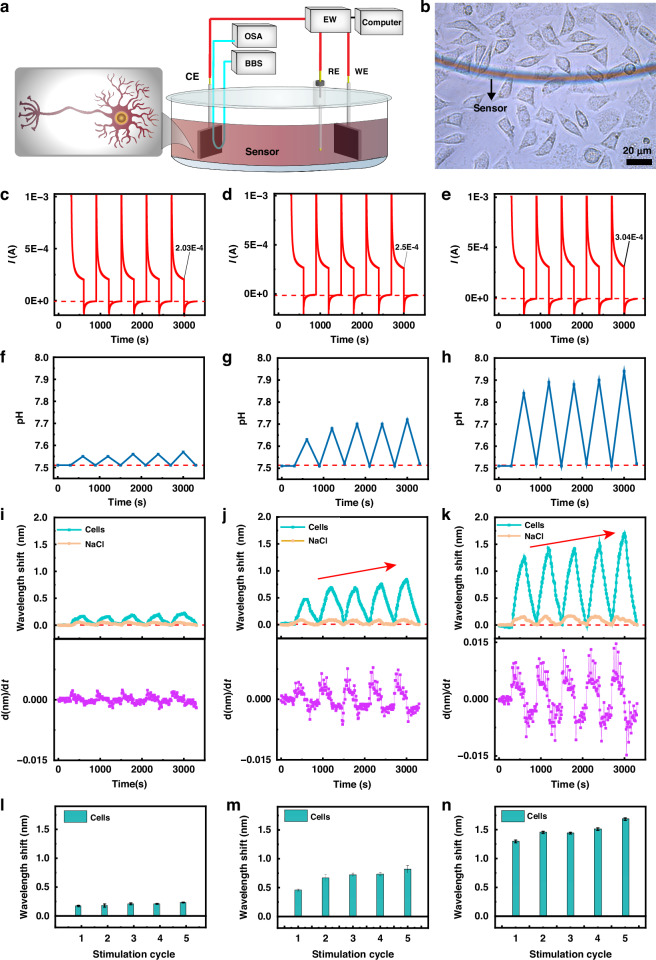
Monitoring of the electrical activity of neurons. **a** Schematic diagram of monitoring of the cell electrification process via the fiber-optic sensor with the GO-MoS_2_-Au interface. The microfiber was positioned at a distance of 204 μm from the counter electrode. (BBS broadband source, OSA optical spectrum analyzer, EW electrochemical workstation, CE counter electrode, RE reference electrode, WE working electrode). **b** Photograph of the placement of the sensor in the cell cluster. **c**, **d**, **e** Electricity generation signal recorded by the electrochemical workstation. **f**, **g**, **h** Changes in the pH during electrical stimulation. **i**, **j**, **k** Wavelength shifts and derivative of the wavelength shift with respect to time d(nm)/d*t* during electrical stimulation. **l**, **m,** **n** Average wavelength shifts obtained from three measurements. (**c**, **f**, **i**, **l**: current was 0.2 mA; **d**, **g**, **j**, **m**: current was 0.25 mA; **e**, **h**, **k**, **n**: current was 0.3 mA)

The ability of the sensor to detect microcurrents enables its application in the study of the conductivity of neuronal cell populations, which reflects the performance and working mechanism of the nervous system, as shown in Fig. 8. The sensing region of the sensor was positioned in a nerve cell population to monitor the microcurrent transmission behavior. The cells were immersed in a sodium chloride solution (0.1 M) that served as their external environment, while a voltage was applied by an electrochemical workstation to establish a microcurrent between adjacent cells (Fig. 8a, b). With the emergence and disappearance of the microcurrent (Fig. 8c–e), the pH at the counter electrode gradually increased when the voltage was applied and gradually reverted when the voltage was removed (Fig. 8f–h). Additionally, distinct redshifts and blueshifts in the spectrum were observed (Fig. 8i–k). Moreover, as the microcurrent increased from 200 to 300 μA, a gradual increase in the wavelength shift from 0.2 nm to 1.5 nm was observed (Fig. 8i–n). Similarly, the velocity peaks of the derivative of the wavelength shift with respect to time were evaluated to investigate the highest rate of change in the wavelength shift during the process of the pulse current appearing and disappearing (Fig. 8i–k). The findings indicate that the acceleration of the wavelength shift velocity was not an instantaneous event but rather a process in which the velocity gradually accelerated to the maximum speed and then gradually decelerated back to the original state. This result is in line with the pH change process in the cell solution and reveals the process of gradual polarization of neuronal cells^47,48^ under the influence of microcurrents, which cannot be discerned only from the current results obtained by the electrochemical workstation. Under the influence of different currents, the speeds (times) at which the wavelength shift reached the maximum and minimum values were similar.

Interestingly, as the number of current cycles increased, the cells exhibited a gradual increase in the microcurrent at identical voltage levels, indicating an increase in the cellular conductivity. This phenomenon was particularly pronounced under higher voltages (Fig. 8m, n), which may be attributed to the facilitation of enhanced alignment of neuronal cell antennae and thus an increase in their conductivity. It has been reported that electrical stimulation can induce depolarization of cytomembranes, change the membrane potential, and affect membrane protein functions such as enzyme activity, membrane‒receptor complexes, and ion-transport channels by altering the charge distribution on biomolecules^47–49^, thereby promoting neural differentiation and regeneration^50^. Thus, the sensor could effectively monitor and quantify both the microcurrents and their respective change rates between cells. Furthermore, it could capture the electrical stimulation process of nerve cells that may not be readily discernible through electrochemical methodologies. In addition, the cell morphology was unaffected by the monitoring process, as evidenced by the photographs taken before and after the process (Fig. S38), indicating that the nerve cells were not damaged by the optical microfiber sensor.

#### Monitoring of hydrogen‒oxygen battery power generation

**Fig. 9:**
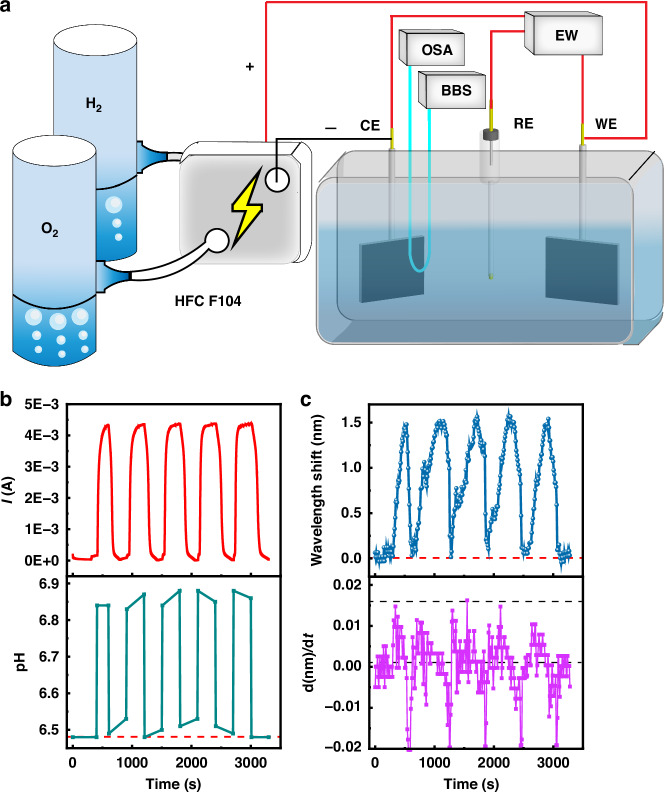
Monitoring of hydrogen‒oxygen battery power generation. **a** Schematic diagram of monitoring of hydrogen‒oxygen battery power generation via the fiber-optic sensor with the GO-MoS_2_-Au interface. The microfiber was positioned at a distance of 204 μm from the counter electrode. (BBS broadband source, OSA optical spectrum analyzer, EW electrochemical workstation, CE counter electrode, RE reference electrode, WE working electrode). **b** Electricity generation signal recorded by an electrochemical workstation and the corresponding pH changes, **c** wavelength shifts, and derivative of the wavelength shift with respect to time d(nm)/d*t*

The current generated by a hydrogen‒oxygen battery serves as a crucial performance indicator, necessitating the utilization of sensors for real-time monitoring of it. The prepared sensor was employed for this purpose (Fig. 9a). For a battery current of approximately 4.3 mA (Fig. 9b), the pH of the electrolyte at the counter electrode rose by approximately 0.36 units (Fig. 9b), leading to the sensor recording a redshift in transmission of approximately 1.5 nm (Fig. 9c). When the battery was deactivated, the pH of the electrolyte and the wavelength shift recorded by the sensor reverted to the original state. When the battery was activated again, the pH and the wavelength shift also changed again, etc. Furthermore, the spectrum demonstrated a maximum instantaneous increase rate when the current reached its peak value (Fig. 9c). Importantly, the direct monitoring of the current by the electrochemical method and the recording of wavelength shifts by our sensor did not adequately capture the changes in the electric flow during multiple battery cycles. However, the derivative of the wavelength shift with respect to time could reveal fluctuations in the battery power generation efficiency across these cycles, making it crucial for evaluating the performance. The results indicated that the rate at which the electrolyte varied in response to the voltage fluctuated with each applied voltage. Hence, this sensor enables real-time monitoring of power generation for hydrogen‒oxygen batteries. Furthermore, it enables evaluation of the subtle disparities in the electricity generation efficiency during each discharge cycle; this crucial information cannot be obtained through an electrochemical workstation.

## Discussion

In this work, we demonstrated the feasibility of using a dielectric fiber-optic sensor to monitor localized proton concentration changes and microcurrents with no interference with the current system operation. The sensor utilized the evanescent field at the micron scale to detect variations in the refractive index of the environment of the sensor induced by changes in the localized proton concentration. The sensor’s response to proton concentration came from the interaction between the oxygen-containing groups (hydroxyl groups) on the microfiber surface and the ions in the electrolyte during electrification. Figures S39, S40 indicate that the greater the number of oxidation groups on the microfiber surface is, the greater the sensitivity of the sensor to changes in the proton concentration. Therefore, in this work, the microfiber after 8 h-oxidation treatment was employed. This sensor enabled microscale insight into the localized proton concentration changes in the electrolyte, thereby facilitating measurement of the corresponding microcurrents. This response stemmed from alterations in the refractive index of the fiber surface, which resulted from interactions between the treated fiber surface and surrounding protons and ions following energization. Consequently, the use of electromagnetic materials can be avoided in the monitoring system.

The dielectric sensor exhibited a limit of detection for a microcurrent of 1 μA and an ideal pH resolution of 0.0052 pH units. The sensing region was a microfiber with a diameter of 8.8 μm. It can capture a greater number of microelectrochemical reaction signals within a detected system on the basis of microscale insight into spatial proton concentration changes, thereby obtaining invaluable information that cannot be obtained through conventional electrochemical methods. This study provides insights into the mechanism of photocurrent attenuation in photogenerated carrier materials during illumination, electrical activation in nerve cells, and fluctuations in the efficiency of electrical energy generation during battery discharge. Since the proton concentration is a fundamental parameter in manipulating many other processes in chemistry and biology, the ability to obtain microscale insights into local proton concentration changes via the sensor provides a powerful complement to electrochemical methods for elucidating microscale reaction mechanisms. Furthermore, the interface enhancement endows the sensor with a heightened sensitivity, facilitates the detection of the subtle proton concentration information generated by smaller microcurrents, and enhances the capacity of the sensor to discern minuscule current variations. The information provided by the prepared dielectric fiber-optic sensor will shed light on proton kinetics, electrochemical and electrobiological mechanisms. It may fill an important gap in the current bioelectricity and battery monitoring methods.

## Materials and methods

### Materials

All chemical reagents were of analytical grade and utilized as received without additional purification. They were supplied by the Sigma-Aldrich Reagent Database Inc. The GO nanosheets and MoS_2_ nanosheets were commercially obtained from XF Nano, Inc. (Nanjing, China). Neuron cells (CP-M207) were purchased from iCell Bioscience, Inc. (Shanghai, China). A bipolar hydrogen‒oxygen‒air fuel cell (F104) was obtained from H-TEC Education Company (USA).

### Instrument

The sensing system consisted of two major subsystems: an optical system for in situ detection and a three-electrode system for calibration. The optical system consisted of a BBS (ASE C + L LIGHT SOURCE, purchased from Golight, China), an optical microfiber sensing probe, and an OSA (IM AQ6370D-02, purchased from YOKOGAWA, Japan). Standard fiber optic connectors were used to connect them sequentially. A platinum electrode was used as the counter electrode and was immersed in the electrolyte. A platinum electrode was used as the counter electrode and was immersed in the electrolyte. A platinum electrode and a calomel electrode were set as the working and reference electrodes, respectively. The optical microfiber sensing probe was attached to the surface of the counter electrode or working electrode. An electrochemical workstation was used to drive the three-electrode system. Both the optical and electrochemical signals were recorded and analyzed by a computer in real time.

### Characterization

The morphology of the microfiber surfaces with various interfaces was observed by SEM (ULTRA 55, ZEISS) and AFM (Bioscope Catalyst Nanoscope-V). The UV‒vis-IR spectra were obtained using a spectrophotometer (Shimadzu, UV-2550). Raman spectroscopy measurements were carried out using a micro-Raman spectrometer (Thermo Fisher Scientific, DXR, excited by a 532 nm laser line). The pH of the electrolyte was measured by a pH meter (FE28-Standard,purchased from METTLER TOLEDO, Switzerland). The microcurrent values of the system was measured by an electrochemical workstation (760E, purchased from purchased from Ch Instruments, China).

### Microfiber fabrication

The tapered microfiber was fabricated by heating a silica single-mode fiber (UVS-INT-PREMIUM, 100536, CorActive High-Tech Inc.) with a 5 mm-wide flame and subsequently stretching it through two linear stages. The geometry of the taper, including its diameter and length in the transition region, was determined by the movement speed of the flame. In the present study, a microfiber with a uniform region and a diameter of 8.8 μm was fabricated.

### Functionalization of the optical microfiber with interfaces

The oxidizer-treated microfiber surface was obtained by immersing the microfiber in a mixture of concentrated sulfuric acid and hydrogen peroxide at a 3:1 volume ratio for 8 h. After that, the microfiber was pulled from the oxidizing solution and rinsed in deionized water to remove the unreacted oxidizing agents on the surface.

The microfiber with interface was fabricated as follows: The microfiber surface was hydroxylated and aminated following the procedure reported in a previous work^34^. Next, the microfiber with amino groups was immersed in a GO dispersion (0.0469 mg/mL) for 30 min, followed by immersion in an APTES solution. Then, the microfiber was immersed in a MoS_2_ dispersion (0.625 mg/mL) for 30 min, followed by immersion in an APTES solution. Finally, the microfiber was functionalized with an Au@Ag_2_S dispersion (0.01 mM) for 10 min and dried. Thus, an optical microfiber with GO-MoS_2_-Au interface was obtained.

The other interfaces on the microfiber undergo the same modifications as described above. For example, by only immersing the microfiber in the GO dispersion, an optical microfiber with GO interface was obtained; by only immersing the microfiber in the MoS_2_ dispersion, an optical microfiber with MoS_2_ interface was obtained; by the sequentially immersing the microfiber in the GO dispersion, APTES solution and MoS_2_ dispersion, an optical microfiber with GO-MoS_2_ interface was obtained.

For the microfiber without interface or the microfibers with interfaces, microfiber could be reused. A single optical microfiber or a single coated microfiber could be used to complete all the detection applications in this work. After the detections, by repeated immersion in deionized water, the surface-adhered ions can be removed, and the microfiber could be reused.

The control optical microfiber shown in Fig. S3 was treated with a sodium hydroxide solution at a concentration of 0.1 M and without treatment with piranha solution.

## Supplementary information

Supporting information

## Data Availability

The data that support the findings of this study are available from the authors upon reasonable request.
