# Supplementary material for: Microscale insight into the proton concentration during electrolytic reaction via an optical microfiber: potential for microcurrent monitoring by a dielectric probe
Source: Light Sci Appl. 2025 Feb 7;14:73. doi: 10.1038/s41377-025-01770-9 (PMC11802907; doi:10.1038/s41377-025-01770-9)
Supplement: Supplementary file 1 — Supporting information [file 41377_2025_1770_MOESM1_ESM.docx]

Supplementary Information for

**Microscale insight into the** **proton concentration during electrolytic reaction via an optical microfiber: potential for microcurrent monitoring by a dielectric probe**

Yunyun Huang^*1,2^, Jiaxuan Liang^1,2^, Haotian Wu,^1,2^ Pengwei Chen^1,2^, Aoxiang Xiao^1,2^, and Bai-Ou Guan^*1,2^

^1^Guangdong Provincial Key Laboratory of Optical Fiber Sensing and Communications, Institute of Photonics Technology, Jinan University, Guangzhou 511143, China

^2^College of Physics & Optoelectronic Engineering, Jinan University, Guangzhou, 510632, China

E-mail: Yunyun Huang, email: [thuangyy6@jnu.edu.cn](mailto:thuangyy6@jnu.edu.cn)

Bai-Ou Guan, email: tguanbo@jnu.edu.cn

**This PDF file includes:**

**Supplementary Fig.s**

**Fig. S1.** Optical microscope photo of the microfiber.

**Fig. S2.** Spectral stability of the sensor without interface in aqueous solution.

**Fig. S3.** pH sensitivity of the optical microfiber sensor without an interface in water (pH=7.0) and added HCl and NaOH solutions to adjust the pH of solution, and the optical microfiber sensor without an interface treated by NaOH solution.

**Fig. S4.** Fourier transform infrared spectra of the optical fiber surfaces untreated and treated with piranha solution.

**Fig. S5**. pH changes in the electrolyte at counter electrode with and without voltage (microcurrent was 2 μA).

**Fig. S6.** Height profiles of the atomic force microscopy images.

**Fig. S7.** Transmission spetrum in the functionalization process of GO-MoS_2_-Au interface.

**Fig. S8.** Distribution statistics of Au@Ag_2_S nanoplates on microfiber surfaces.

**Fig. S9.** Finite-difference time-domain mapping of the optical microfiber surfaces with Au@Ag_2_S interface.

**Fig. S10**. Simulation models of the transverse electric field amplitude distributions of the HE_12_ mode.

**Fig. S11.** Bulk refractive index sensitivities of the microfibers with various interfaces..

**Fig. S12.** Transverse electric field amplitude distributions of the HE_12_ mode of microfiber with 10 nm-GO interface and microfiber with 10 nm-MoS_2_ interface calculated via numerical mode simulation software.

**Fig. S13.** SEM images of the microfiber surface with GO interface and the microfiber surface with MoS_2_ interface, and the obtained spectra when the coating solutions were at concentrations 5-fold higher than those used in this work.

**Fig. S14.** SEM image of the microfiber surface with Au@Ag_2_S interface and the obtained spectra when the coating solutions were at concentrations 3-fold higher than that used in this work.

**Fig. S15.** Transmission spetrum when the microfiber with GO-MoS_2_-Au interface at working electrode under microcurrent of 3 µA.

**Fig. S16**. Transmission spetrum when the microfiber with GO-MoS_2_-Au interface at working electrode under microcurrent of 0.25 mA.

**Fig. S17**. Transmission spetrum when the microfiber with GO-MoS_2_ interface at working electrode under microcurrent of 3 µA.

**Fig. S18**. Transmission spetrum when the microfiber with GO-MoS_2_ interface at working electrode under microcurrent of 0.25 mA.

**Fig. S19**. Transmission spetrum when the microfiber with MoS_2_-GO interface at working electrode under microcurrent of 3 µA.

**Fig. S20**. Transmission spetrum when the microfiber with MoS_2_-GO interface at working electrode under microcurrent of 0.25 mA.

**Fig. S21**. Transmission spetrum when the microfiber with MoS_2_ interface at working electrode under microcurrent of 3 µA.

**Fig. S22**. Transmission spetrum when the microfiber with MoS_2_ interface at working electrode under microcurrent of 0.25 mA.

**Fig. S23**. Transmission spetrum when the microfiber with GO interface at working electrode under microcurrent of 3 µA.

**Fig. S24**. Transmission spetrum when the microfiber with GO interface at working electrode under microcurrent of 0.25 mA.

**Fig. S25.** Transmission spetrum when the microfiber with GO-MoS_2_-Au interface was at the counter electrode under microcurrent of 3 µA.

**Fig. S26.** Transmission spetrum when the microfiber with GO-MoS_2_-Au interface was at the counter electrode under microcurrent of 0.25 mA.

**Fig. S27.** Transmission spetrum when the microfiber with GO-MoS_2_ interface was at the counter electrode under microcurrent of 3 µA.

**Fig. S28.** Transmission spetrum when the microfiber with GO-MoS_2_ interface was at the counter electrode under microcurrent of 0.25 mA.

**Fig. S29.** Transmission spetrum when the microfiber with MoS_2_-GO interface was at the counter electrode under microcurrent of 3 µA.

**Fig. S30.** Transmission spetrum when the microfiber with MoS_2_-GO interface was at the counter electrode under microcurrent of 0.25 mA.

**Fig. S31.** Transmission spetrum when the microfiber with MoS_2_ interface was at the counter electrode under microcurrent of 3 µA.

**Fig. S32.** Transmission spetrum when the microfiber with MoS_2_ interface was at the counter electrode under microcurrent of 0.25 mA.

**Fig. S33.** Transmission spetrum when the microfiber with GO interface was at the counter electrode under microcurrent of 3 µA.

**Fig. S34.** Transmission spetrum when the microfiber with GO interface was at the counter electrode under microcurrent of 0.25 mA.

**Fig. S35.** Transmission spetrum when the microfiber with GO-MoS_2_-Au interface was at the counter electrode under microcurrent increasing from 3μA to 0.25 mA.

**Fig. S36**. Wavelength shifts recorded by the sensor with the GO-MoS_2_-Au interface at the counter electrode when microcurrent was 2 μA.

**Fig. S37.** Electrochemical curve of 1 μA and wavelength shifts recorded by the sensor with the GO-MoS_2_-Au interface at the counter electrode .

**Fig. S38.** Optical photographs of cells before and after detection by the sensor with GO-MoS_2_-Au interface.

**Fig. S39**. O 1s X-ray photoelectron spectroscopy (XPS) spectra of the microfibers after being treated with the same oxidant for various periods of time.

**Fig. S40**. Wavelength shift of the microfiber sensor without interface responding to a 0.25 mA-current at the counter electrode depending on the surface oxidation degree.

**Tables**

**Table S1** Distribution statistics of Au@Ag_2_S nanoplates calculated from Fig. S8.


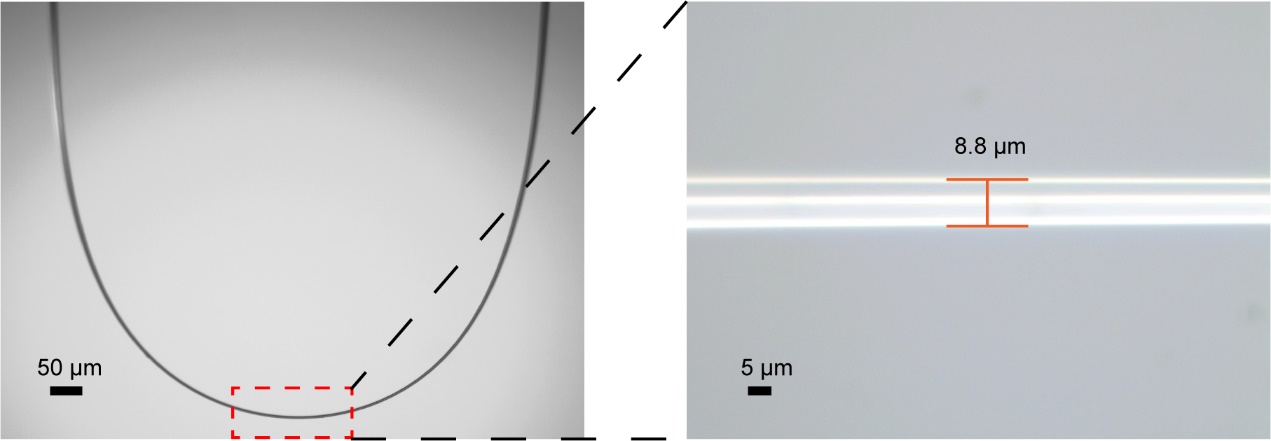


**Fig. S1**. Optical microscope photo of the microfiber.


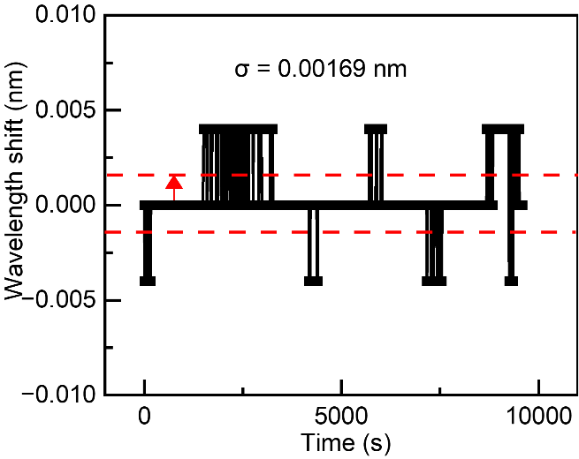


**Fig. S2.** Spectral stability of the sensor without interface in aqueous solution.


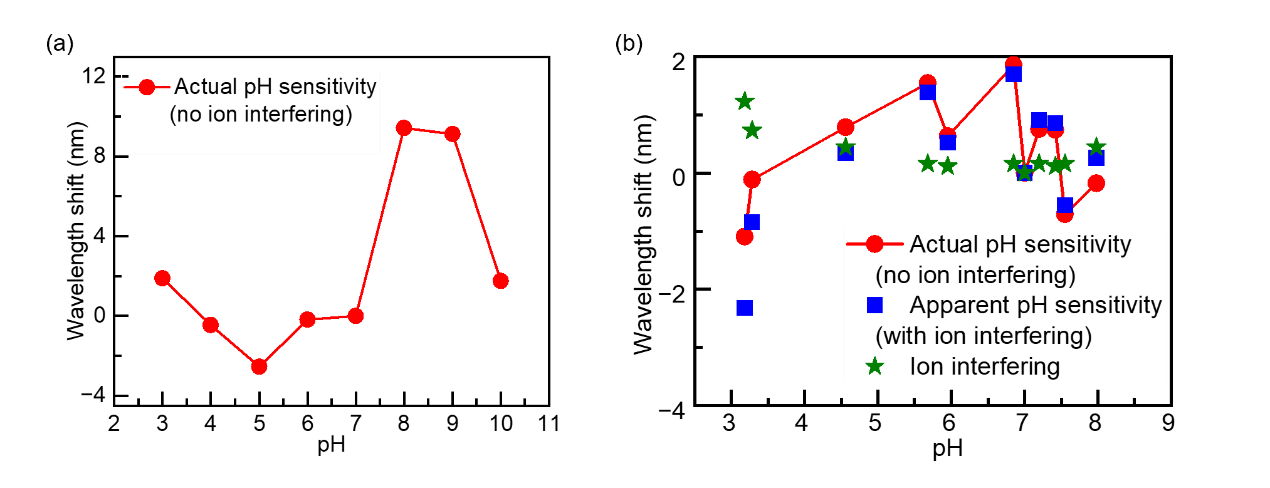


**Fig. S3.** pH sensitivity of (a) the optical microfiber sensor without an interface in water (pH=7.0) and added HCl and NaOH solutions to adjust the pH of solution, and (b) the optical microfiber sensor without an interface treated by NaOH solution.


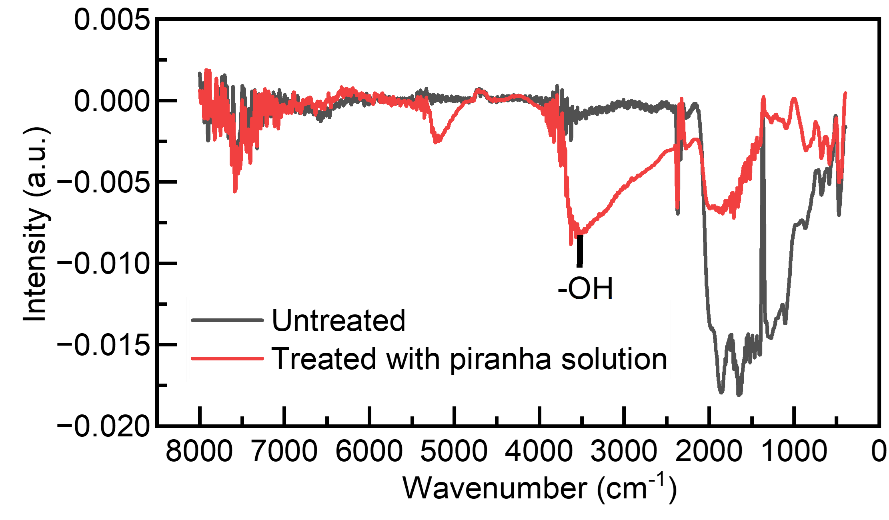


**Fig. S4.** Fourier transform infrared spectra of the optical fiber surfaces untreated and treated with piranha solution.


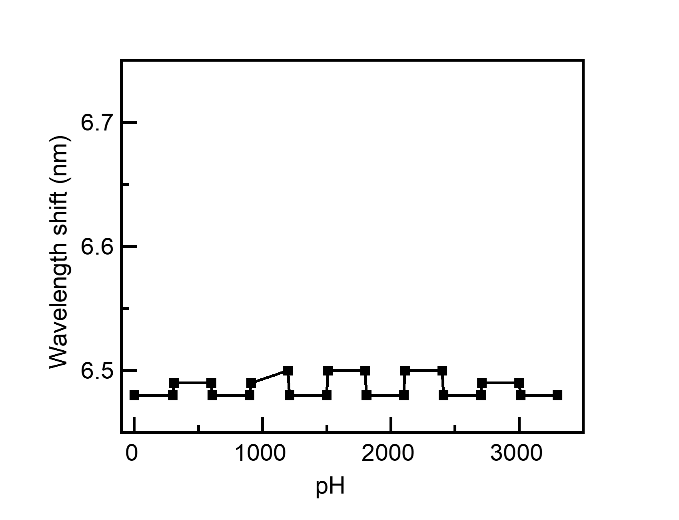


**Fig. S5**. pH changes in the electrolyte at counter electrode with and without voltage (microcurrent was 2 μA).


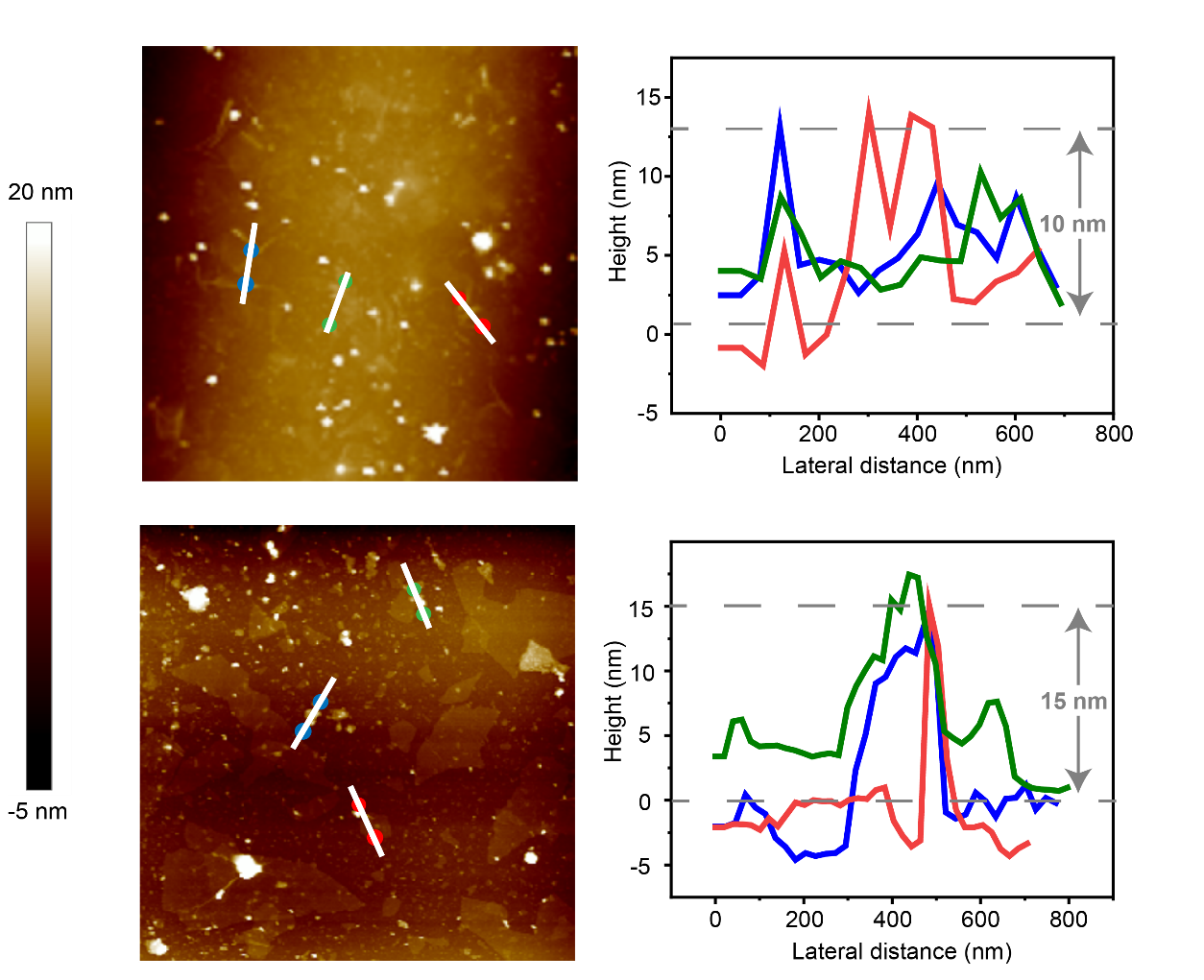


**Fig. S6**. Height profiles of the atomic force microscopy (AFM) images of fiber surfaces with (a) GO-MoS_2_ and (b) MoS_2_-GO. (Due to the limitations of AFM resolution, microfibers could not be used. Instead, the same processed optical fibers with diameter of 125 μm were used for measurement. The results can provide a reference for the thickness of the surface layer on the microfiber.)


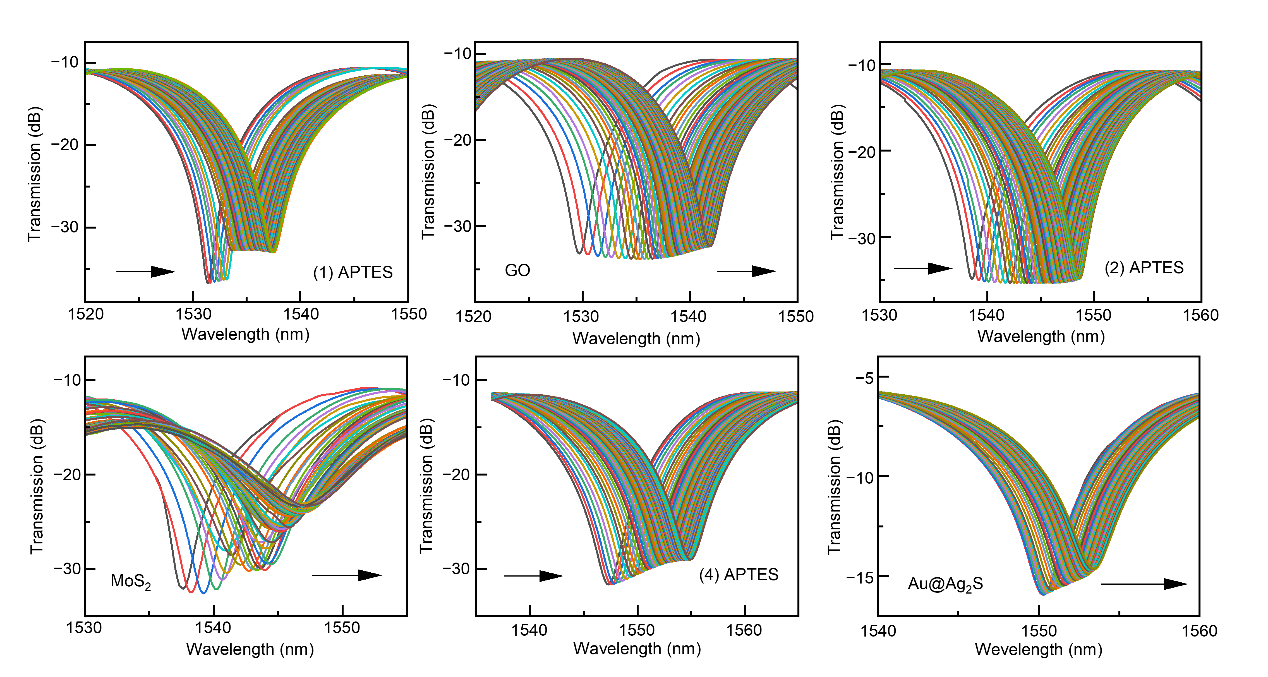


**Fig. S7**. Transmission spetrum in the functionalization process of GO-MoS_2_-Au interface. (Spectrum was recorded at an interval of 30 s along the arrow direction.)


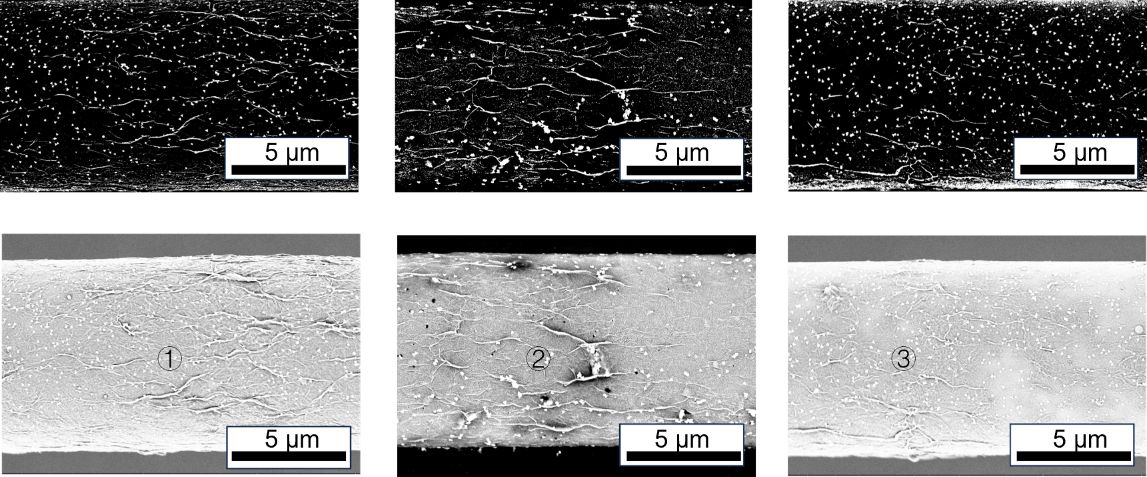


**Fig. S8**. Distribution statistics of Au@Ag_2_S nanoplates on microfiber surfaces.

**Table S1**. Distribution statistics of Au@Ag_2_S nanoplates calculated from Fig. S8

|  | % |
| --- | --- |
| ① | 4.92 |
| ② | 5.17 |
| ③ | 8.91 |
| Average | 6.33±1.82 |


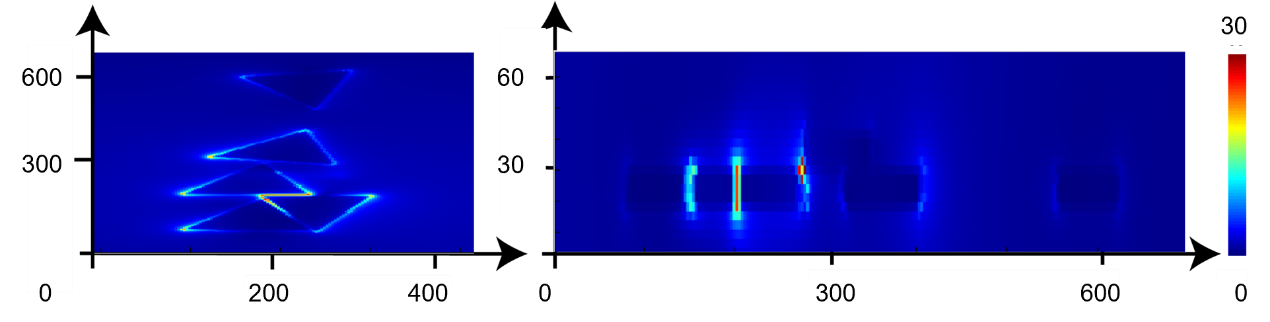


**Fig. S9**. Finite-difference time-domain (FDTD) mapping of the optical microfiber surfaces with Au@Ag_2_S interface.


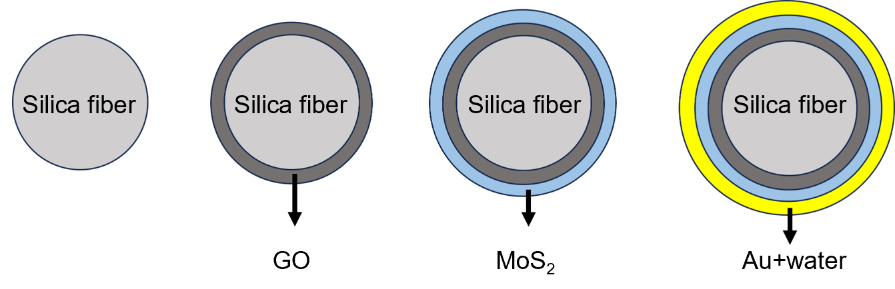


**Fig. S10**. Simulation models of the transverse electric field amplitude distributions of the HE_12_ mode.


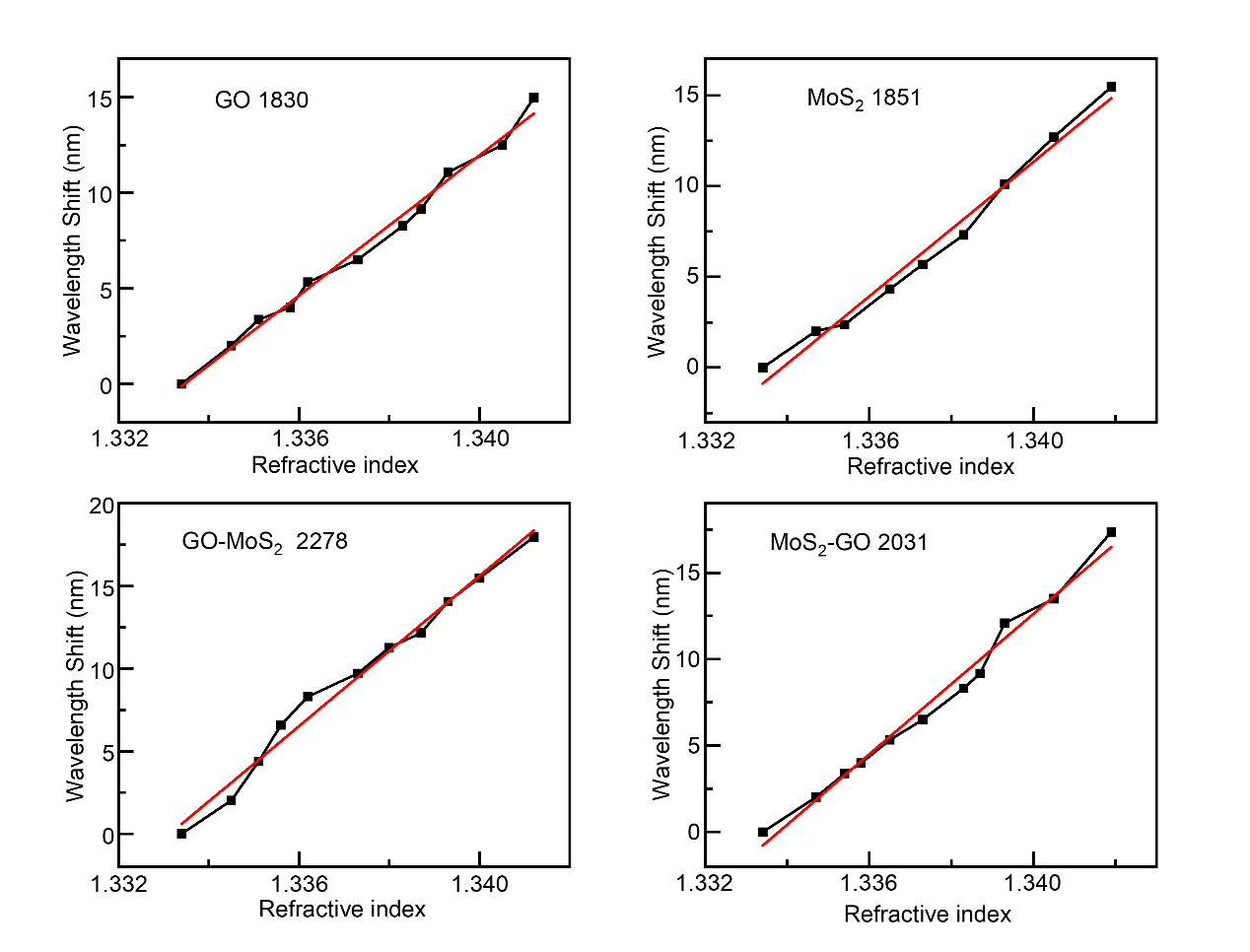


**Fig. S11**. Bulk refractive index sensitivities of the microfibers with various interfaces.


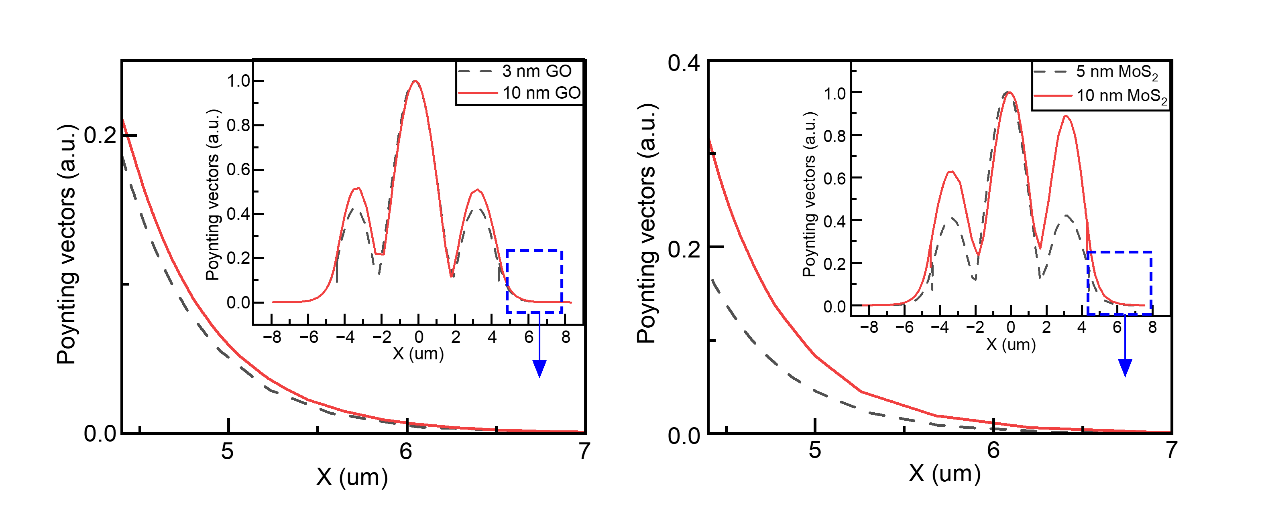


**Fig. S12.** Transverse electric field amplitude distributions of the HE_12_ mode of microfiber with 10 nm-GO interface and microfiber with 10 nm-MoS_2_ interface calculated via numerical mode simulation software. (The thicknesses of 3 nm for GO and 5 nm for MoS_2_ were used in our work.)


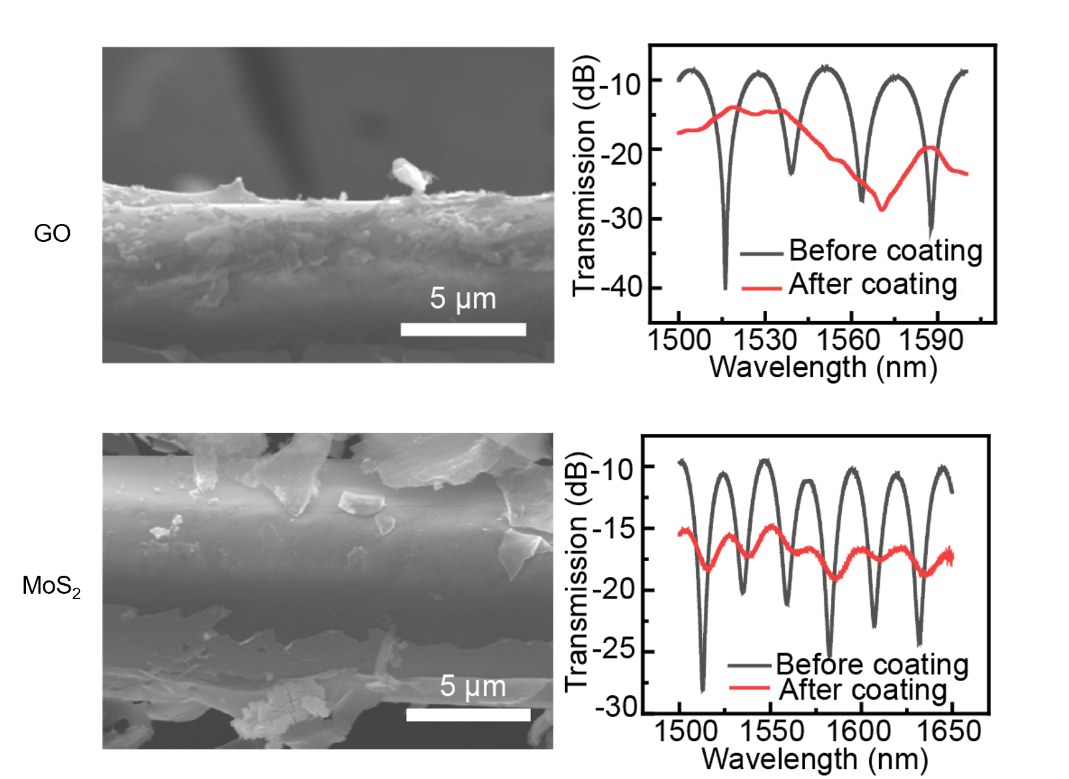


**Fig. S13.** SEM images of the microfiber surface with GO interface and the microfiber surface with MoS_2_ interface, and the obtained spectra when the coating solutions were at concentrations 5-fold higher than those used in our work.


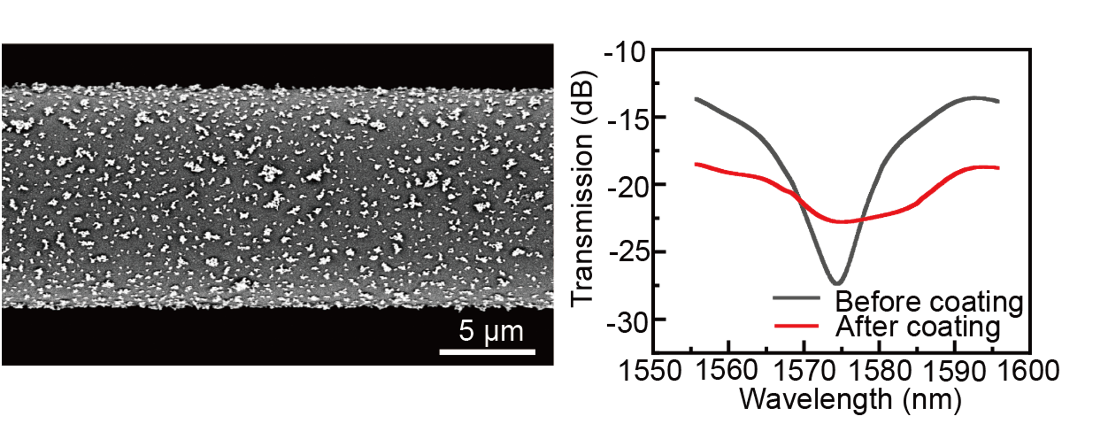


**Fig. S14.** SEM image of the microfiber surface with Au@Ag_2_S interface and the obtained spectra when the coating solutions were at concentrations 3-fold higher than that used in our work.


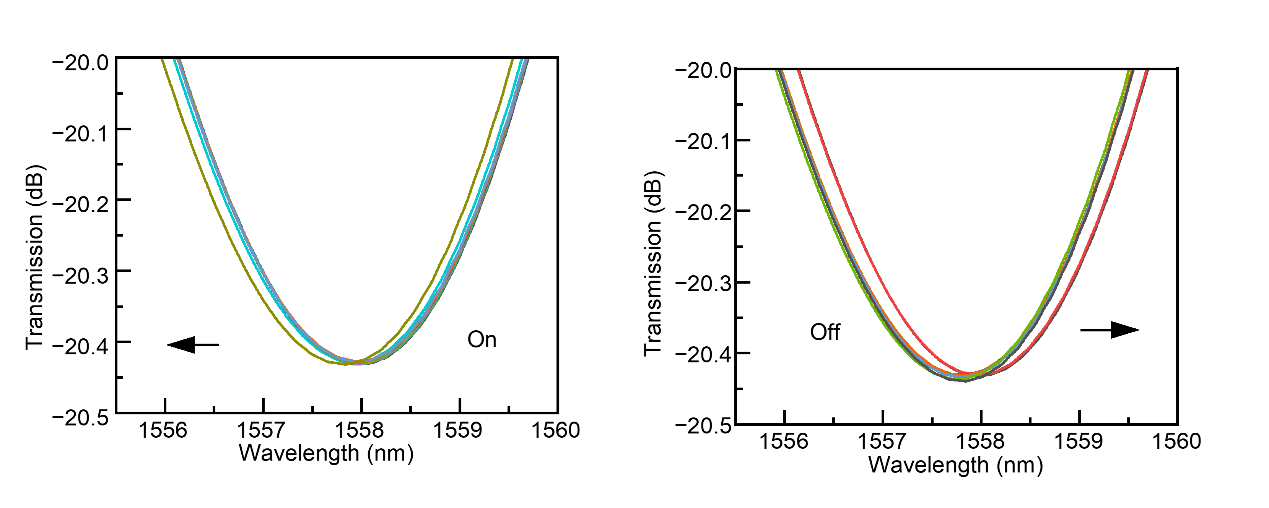


**Fig. S15**. Transmission spetrum when the microfiber with GO-MoS_2_-Au interface at working electrode under microcurrent of 3 µA. (Spectrum was recorded at an interval of 10 s along the arrow direction.)


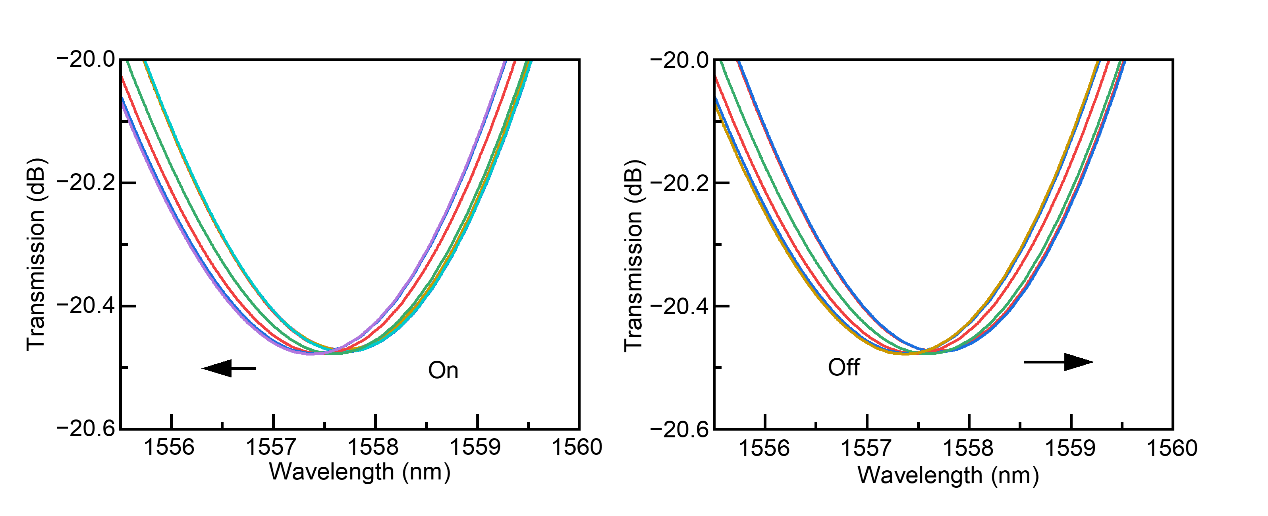


**Fig. S16**. Transmission spetrum when the microfiber with GO-MoS_2_-Au interface at working electrode under microcurrent of 0.25 mA. (Spectrum was recorded at an interval of 10 s along the arrow direction.)


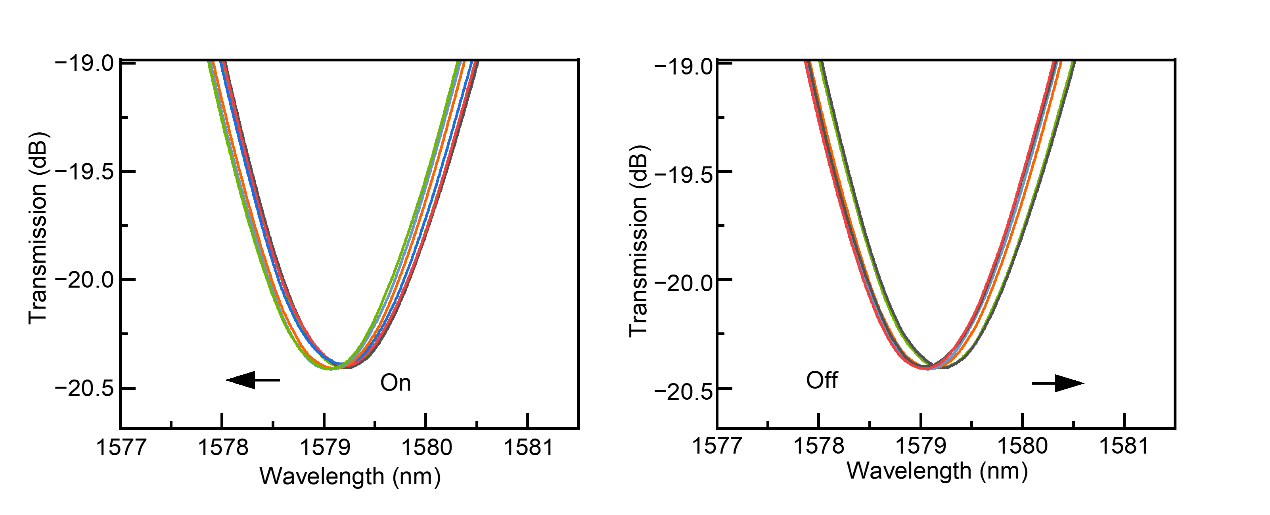


**Fig. S17**. Transmission spetrum when the microfiber with GO-MoS_2_ interface at working electrode under microcurrent of 3 µA. (Spectrum was recorded at an interval of 10 s along the arrow direction.)


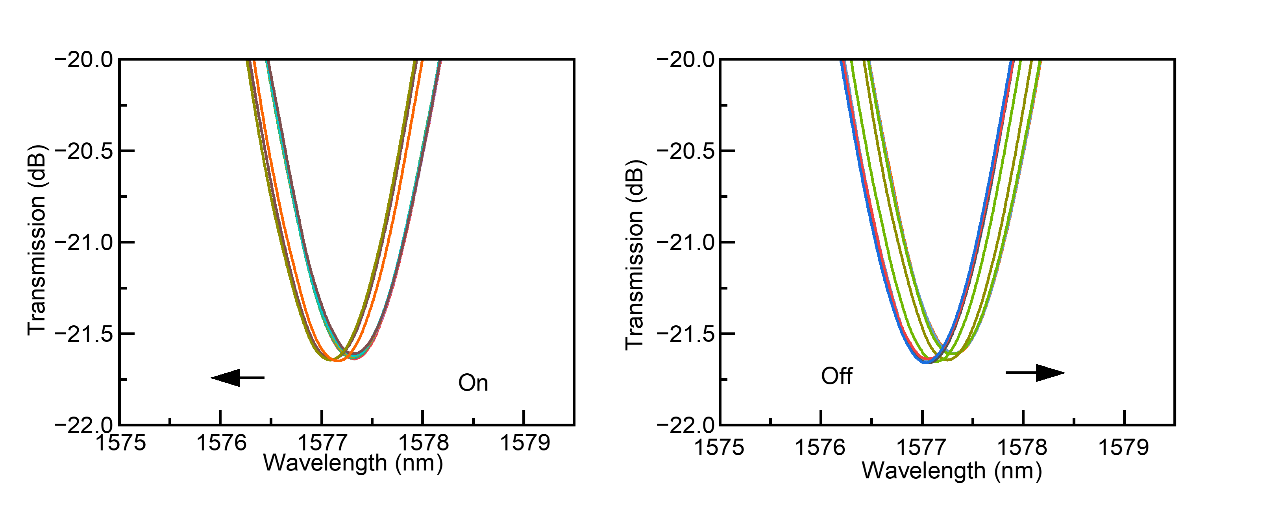


**Fig. S18**. Transmission spetrum when the microfiber with GO-MoS_2_ interface at working electrode under microcurrent of 0.25 mA. (Spectrum was recorded at an interval of 10 s along the arrow direction.)


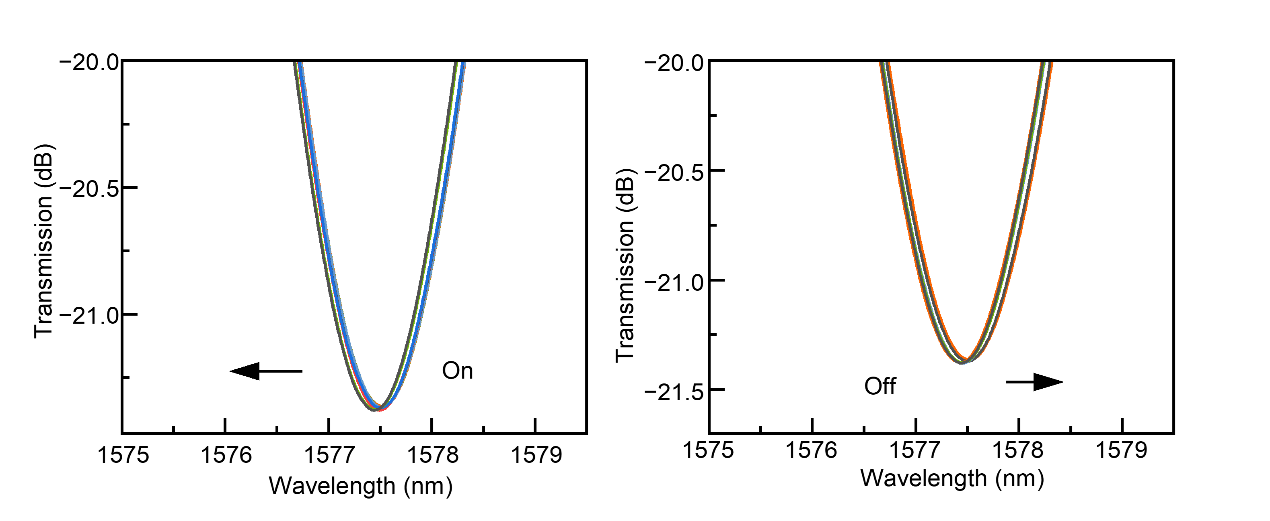


**Fig. S19**. Transmission spetrum when the microfiber with MoS_2_-GO interface at working electrode under microcurrent of 3 µA. (Spectrum was recorded at an interval of 10 s along the arrow direction.)


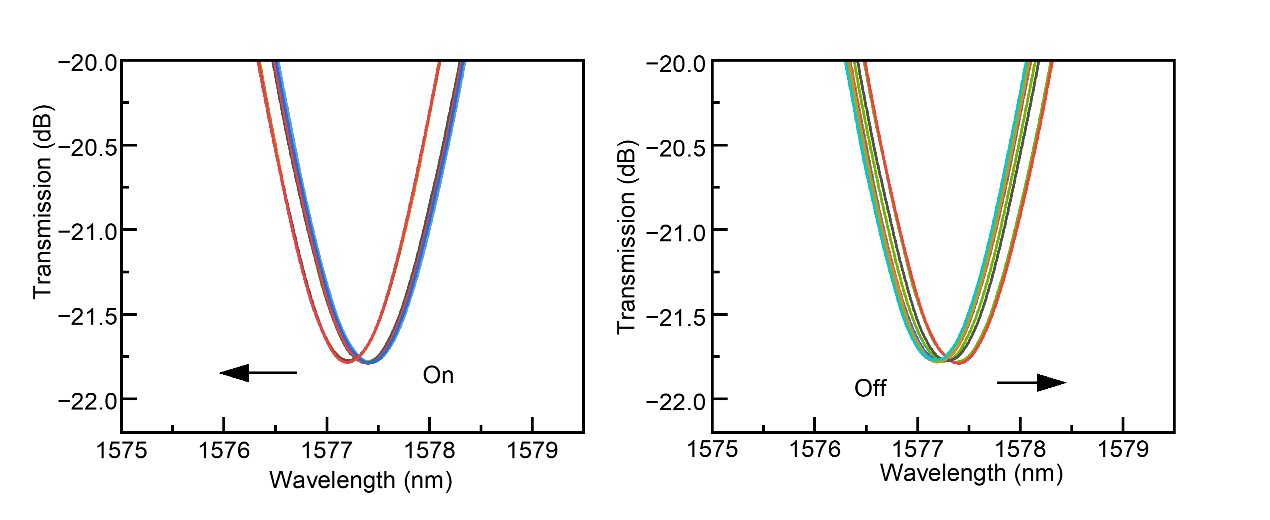


**Fig. S20**. Transmission spetrum when the microfiber with MoS_2_-GO interface at working electrode under microcurrent of 0.25 mA. (Spectrum was recorded at an interval of 10 s along the arrow direction.)


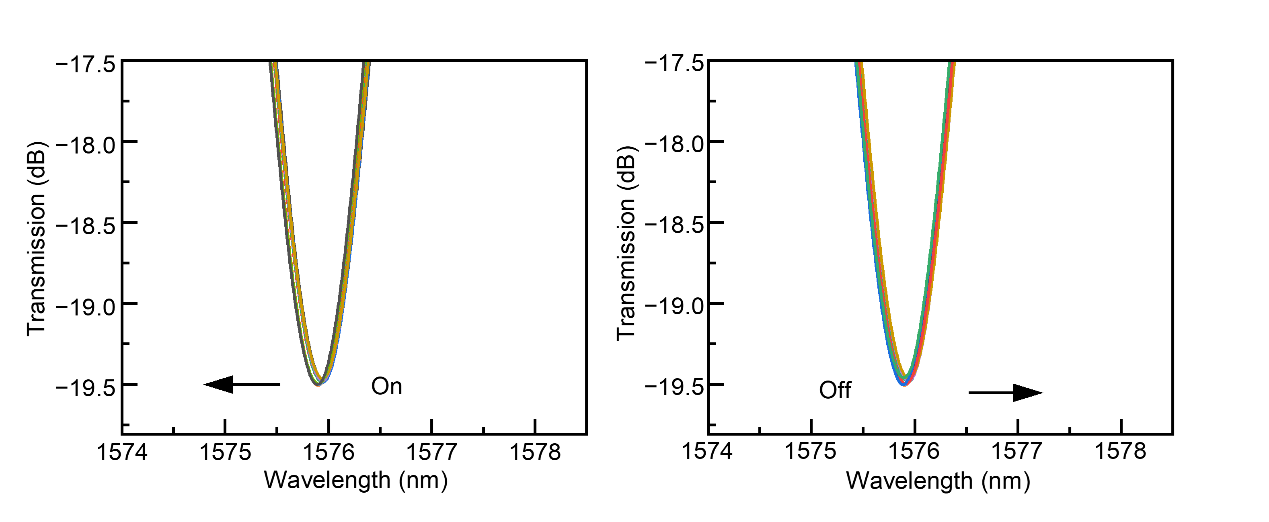


**Fig. S21**. Transmission spetrum when the microfiber with MoS_2_ interface at working electrode under microcurrent of 3 µA. (Spectrum was recorded at an interval of 10 s along the arrow direction.)


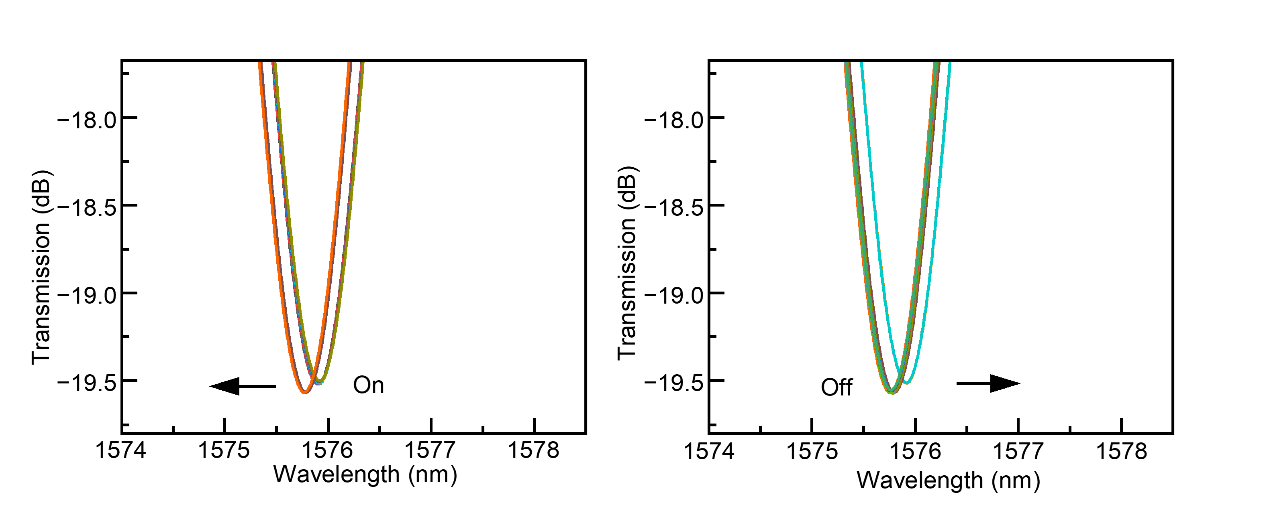


**Fig. S22**. Transmission spetrum when the microfiber with MoS_2_ interface at working electrode under microcurrent of 0.25 mA. (Spectrum was recorded at an interval of 10 s along the arrow direction.)


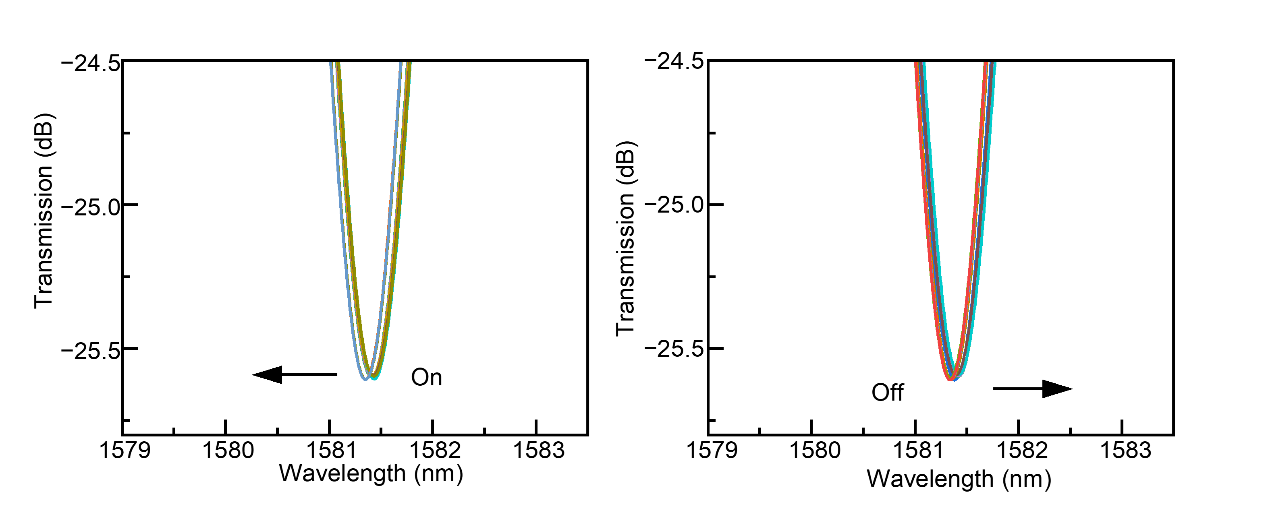


**Fig. S23**. Transmission spetrum when the microfiber with GO interface at working electrode under microcurrent of 3 µA. (Spectrum was recorded at an interval of 10 s along the arrow direction.)


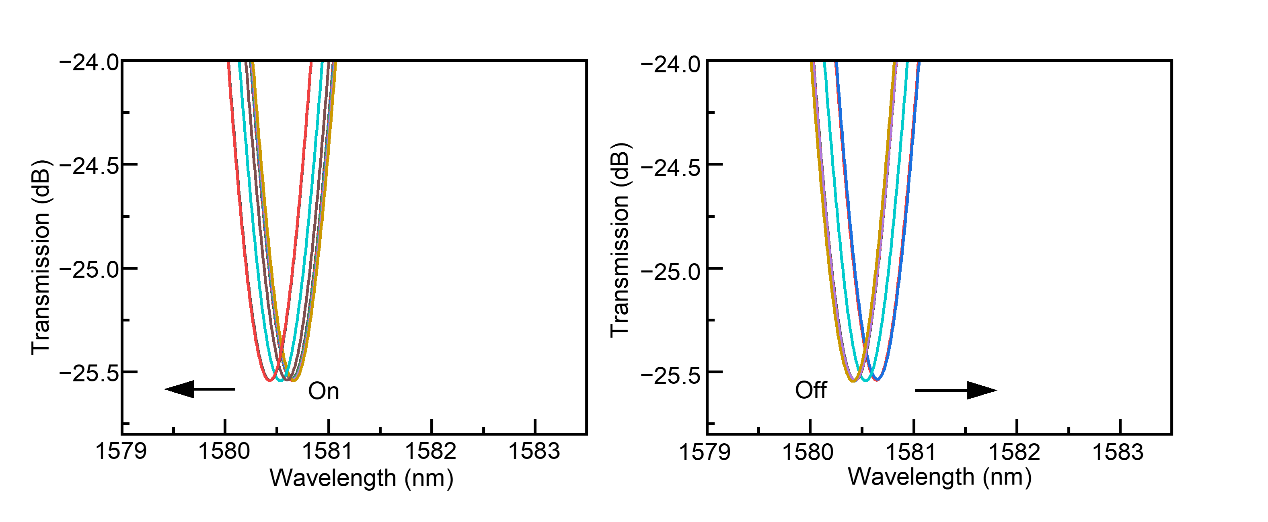


**Fig. S24**. Transmission spetrum when the microfiber with GO interface at working electrode under microcurrent of 0.25 mA. (Spectrum was recorded at an interval of 10 s along the arrow direction.)


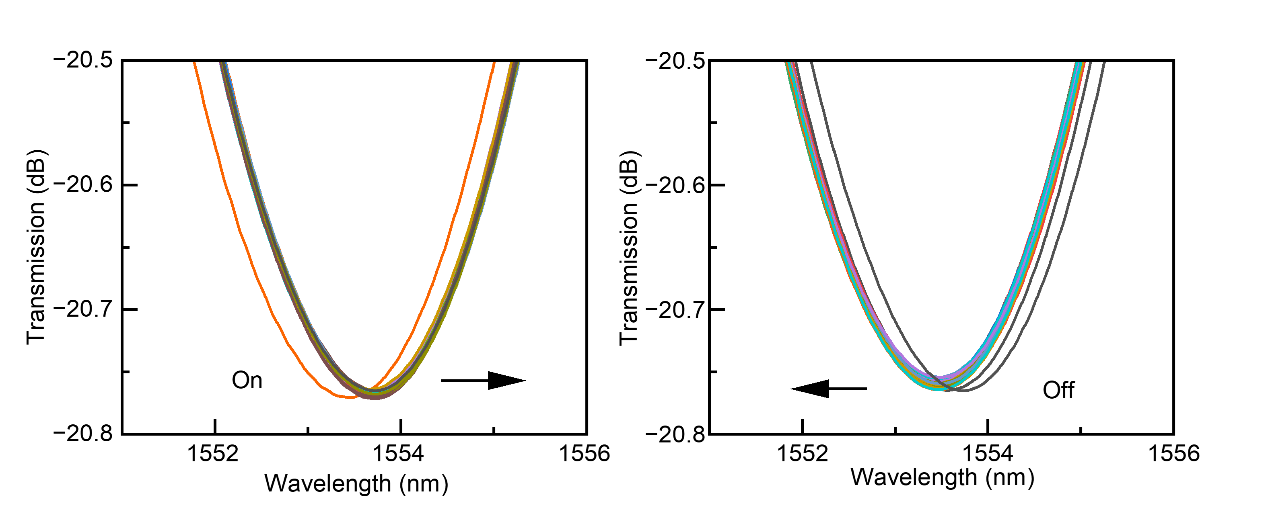


**Fig. S25**. Transmission spetrum when the microfiber with GO-MoS_2_-Au interface at the counter electrode under microcurrent of 3 µA. (Spectrum was recorded at an interval of 5 s along the arrow direction.)


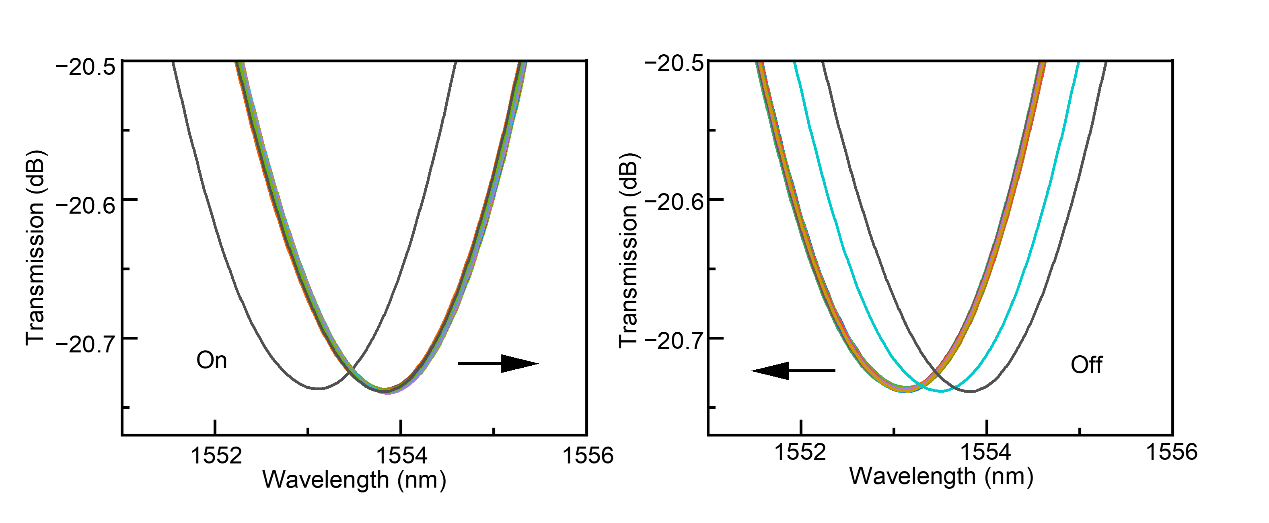


**Fig. S26**. Transmission spetrum when the microfiber with GO-MoS_2_-Au interface at the counter electrode under microcurrent of 0.25 mA. (Spectrum was recorded at an interval of 5 s along the arrow direction.)


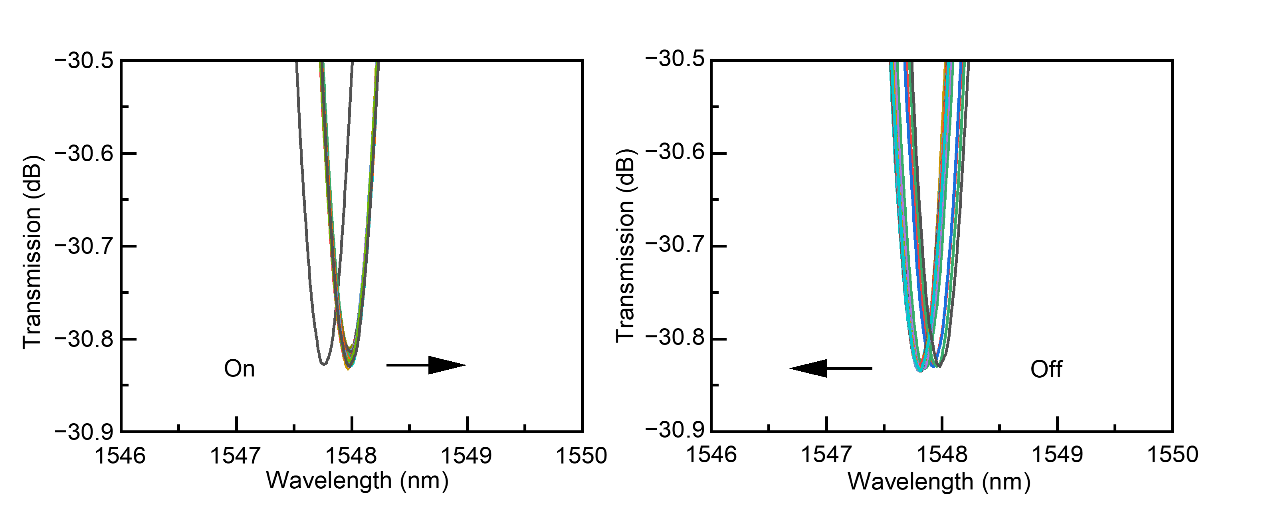


**Fig. S27**. Transmission spetrum when the microfiber with GO-MoS_2_ interface at the counter electrode under microcurrent of 3 µA. (Spectrum was recorded at an interval of 5 s along the arrow direction.)


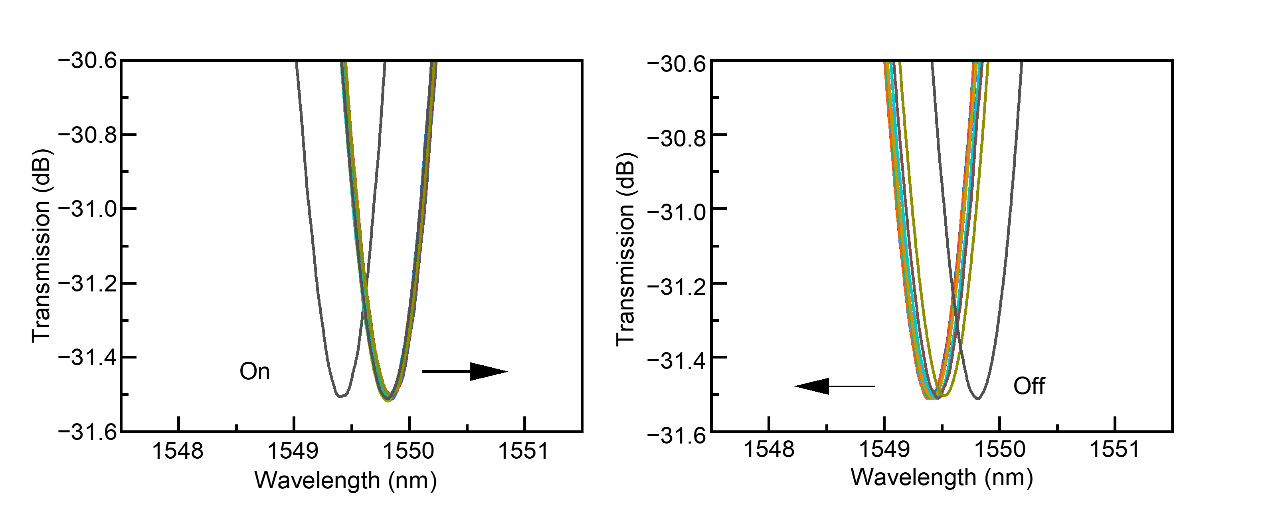


**Fig. S28**. Transmission spetrum when the microfiber with GO-MoS_2_ interface at the counter electrode under microcurrent of 0.25 mA. (Spectrum was recorded at an interval of 5 s along the arrow direction.)


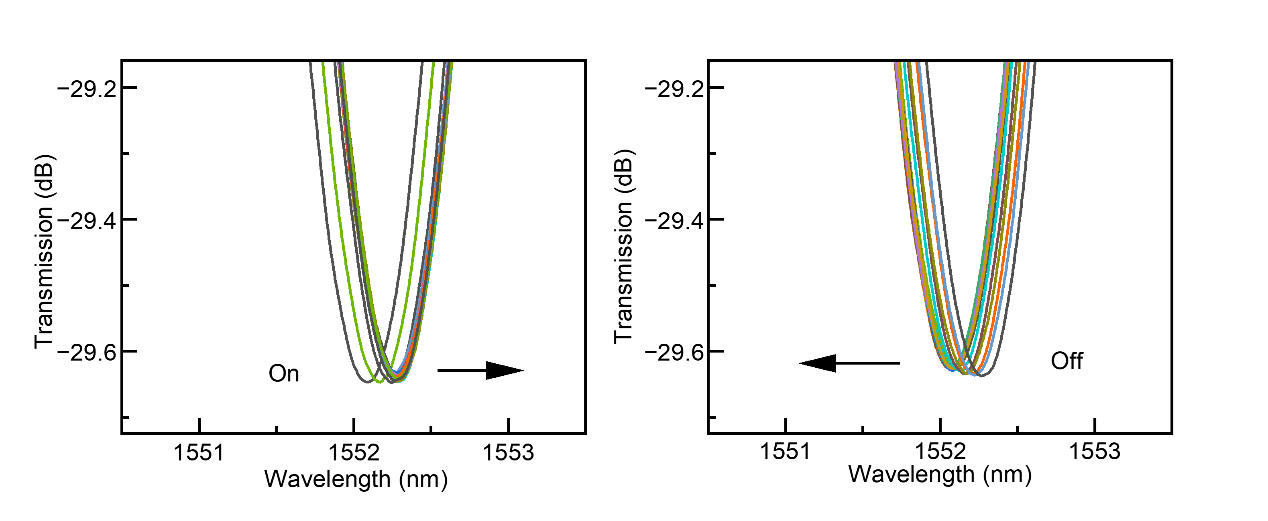


**Fig. S29**. Transmission spetrum when the microfiber with MoS_2_-GO interface at the counter electrode under microcurrent of 3 µA. (Spectrum was recorded at an interval of 5 s along the arrow direction.)


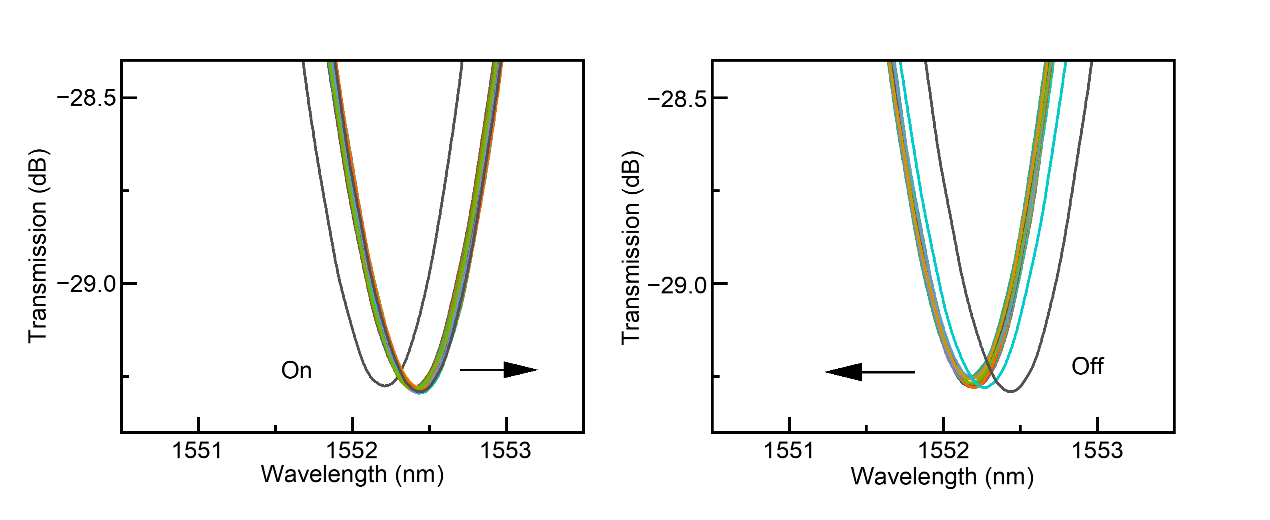


**Fig. S30**. Transmission spetrum when the microfiber with MoS_2_-GO interface at the counter electrode under microcurrent of 0.25 mA. (Spectrum was recorded at an interval of 5 s along the arrow direction.)


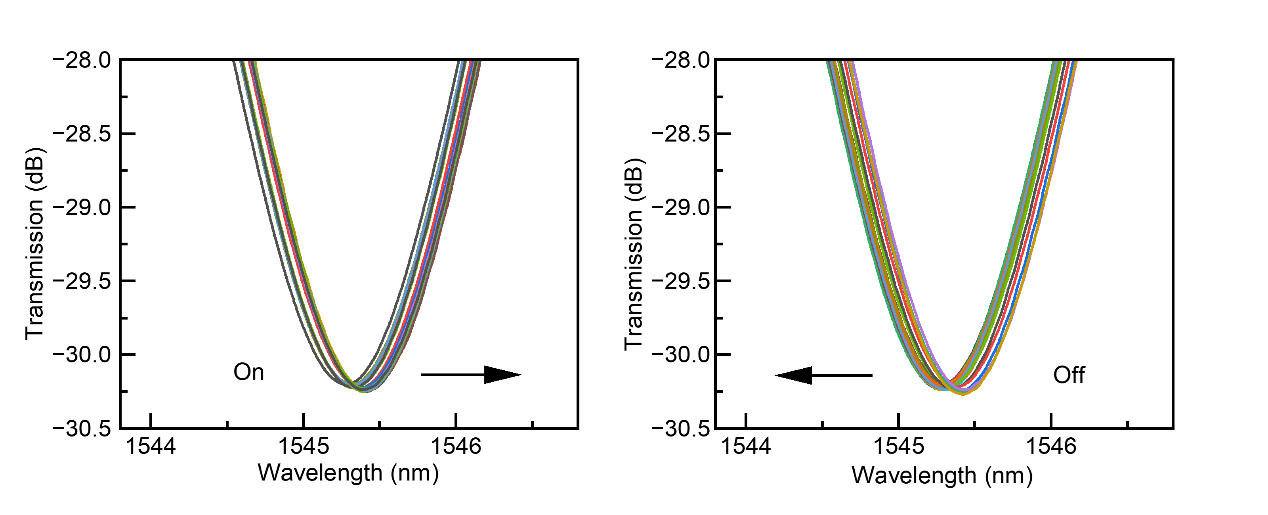


**Fig. S31**. Transmission spetrum when the microfiber with MoS_2_ interface at the counter electrode under microcurrent of 3 µA. (Spectrum was recorded at an interval of 5 s along the arrow direction.)


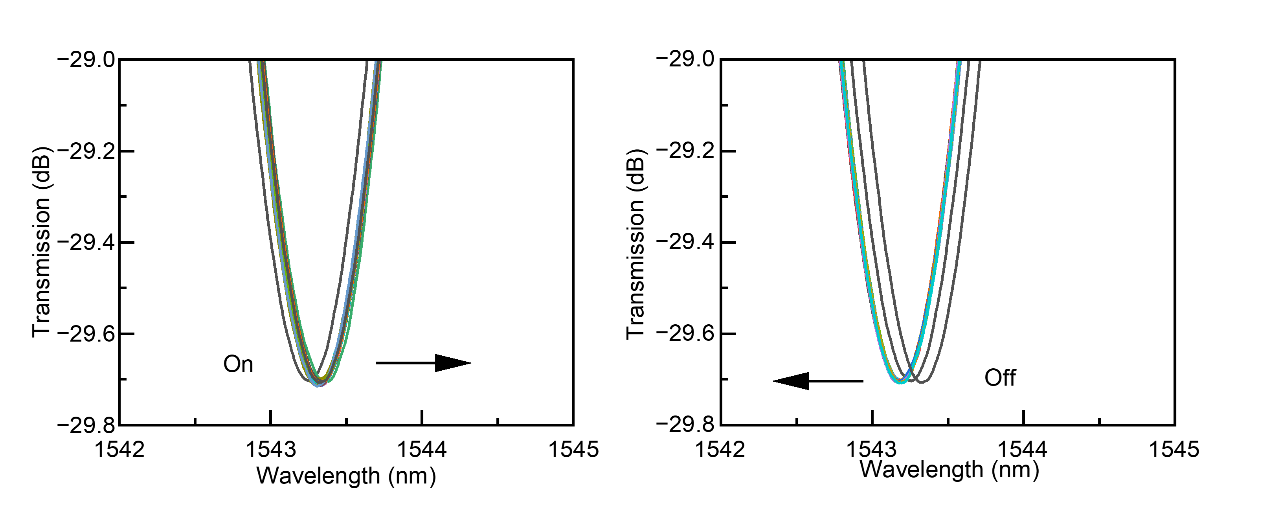


**Fig. S32**. Transmission spetrum when the microfiber with MoS_2_ interface at the counter electrode under microcurrent of 0.25 mA. (Spectrum was recorded at an interval of 5 s along the arrow direction.)


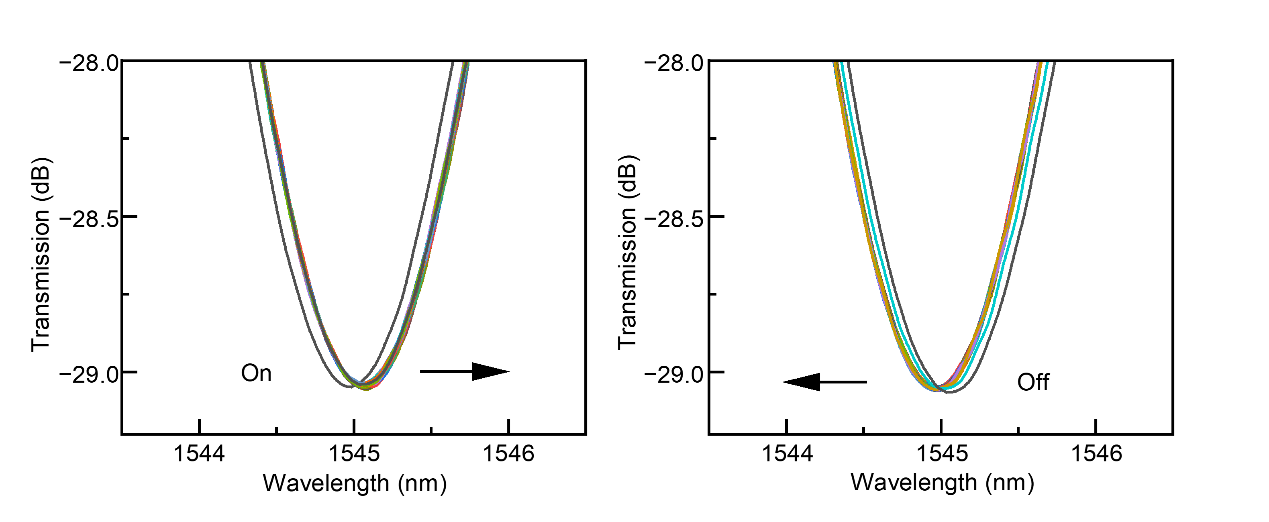


**Fig. S33**. Transmission spetrum when the microfiber with GO interface at the counter electrode under microcurrent of 3 µA. (Spectrum was recorded at an interval of 5 s along the arrow direction.)


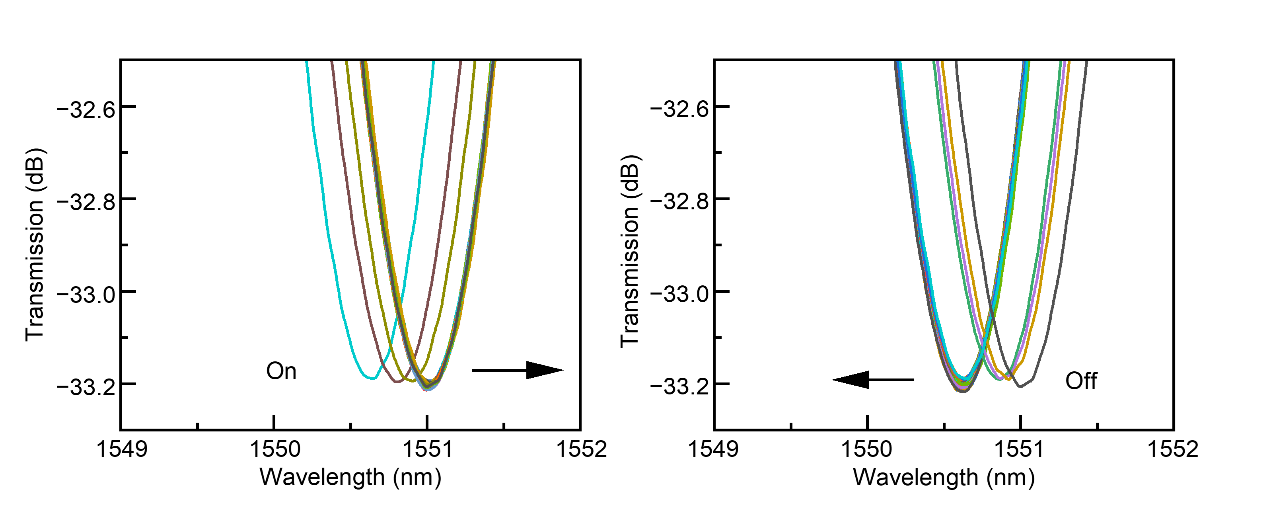


**Fig. S34**. Transmission spetrum when the microfiber with GO interface at the counter electrode under microcurrent of 0.25 mA. (Spectrum was recorded at an interval of 5 s along the arrow direction.)


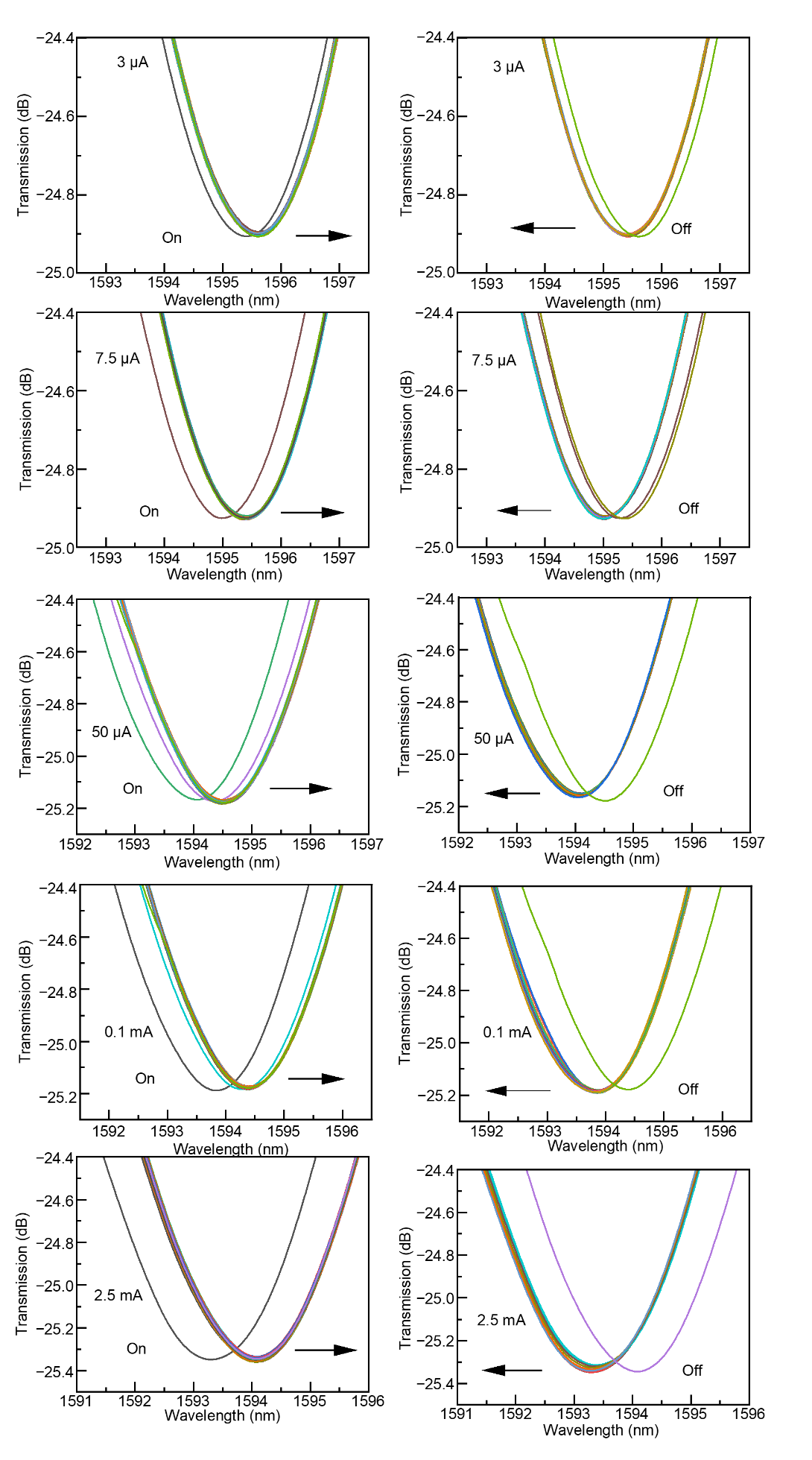


**Fig. S35**. Transmission spetrum when the microfiber with GO-MoS_2_-Au interface was at the counter electrode under microcurrent increasing from 3μA to 0.25 mA.


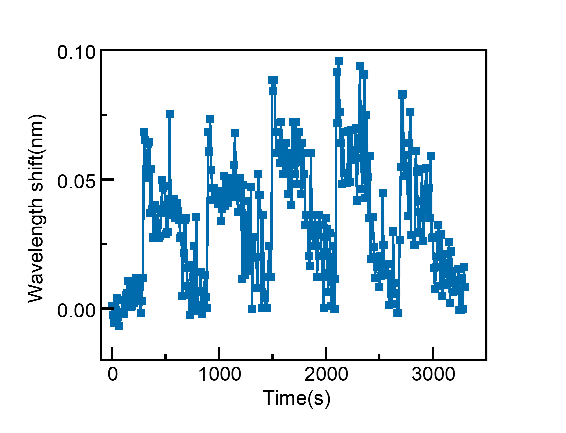


**Fig. S36**. Wavelength shifts recorded by the sensor with the GO-MoS_2_-Au interface at the counter electrode when microcurrent was 2 μA.


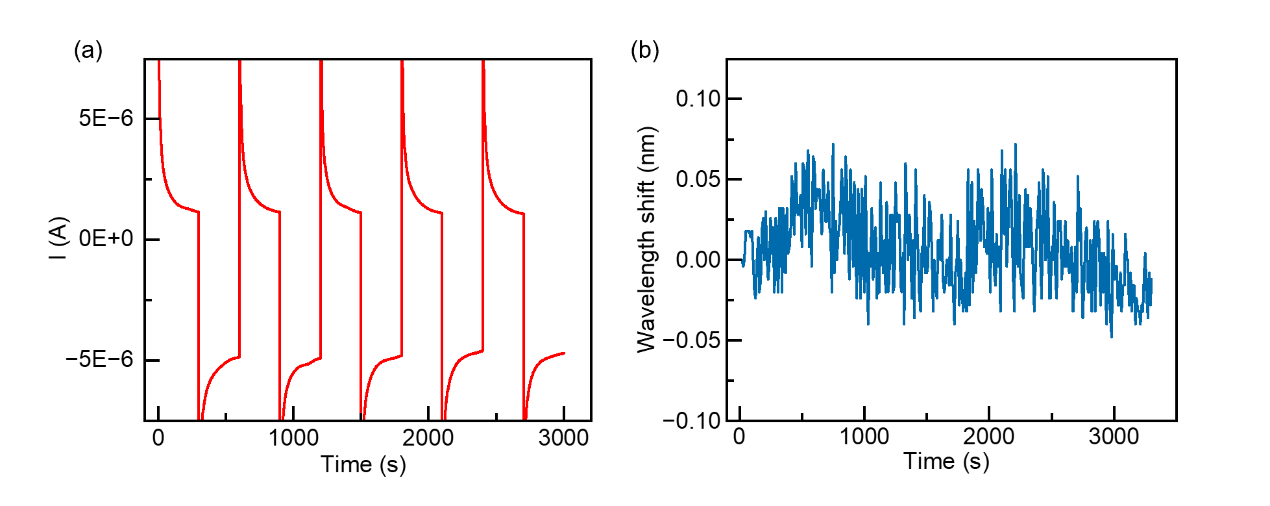


**Fig. S37.** (a) Electrochemical curve of 1 μA and (b) wavelength shifts recorded by the sensor with the GO-MoS_2_-Au interface at the counter electrode .


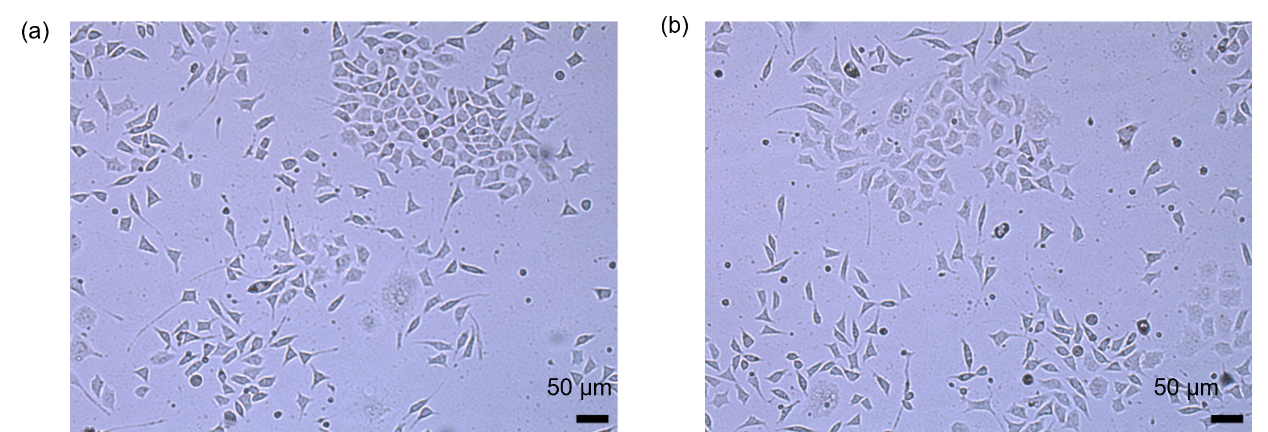


**Fig. S38**. Optical photographs of cells (a) before and (b) after detection by the sensor with GO-MoS_2_-Au interface.


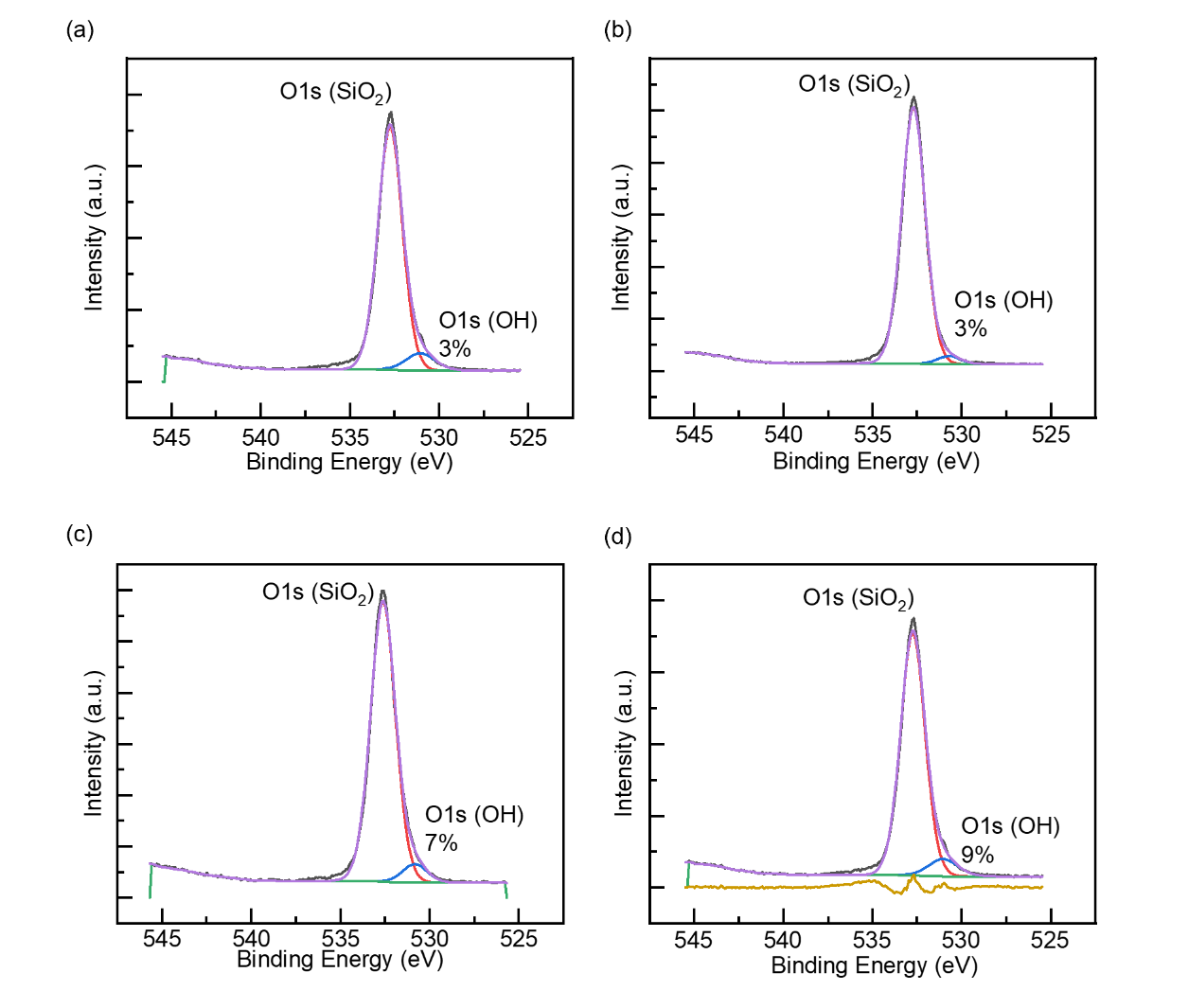


**Fig. S39**. O 1s X-ray photoelectron spectroscopy (XPS) spectra of the microfibers after being treated with the same oxidant for various periods of time. (a) Microfiber without oxidant treatment. (OH/SiO_2_ ratio: 3%.) (b) Microfiber with oxidant treatment for 10 min. (OH/SiO_2_ ratio: 3%.) (c) Microfiber with oxidant treatment for 4 h. (OH/SiO_2_ ratio: 7%.) (d) Microfiber with oxidant treatment for 8 h. (OH/SiO_2_ ratio: 9%.) The O in SiO_2_ comes from the entire solid microfiber, so the proportion is very high; the OH content is relatively low because OH is only distributed on the surface of the microfiber.


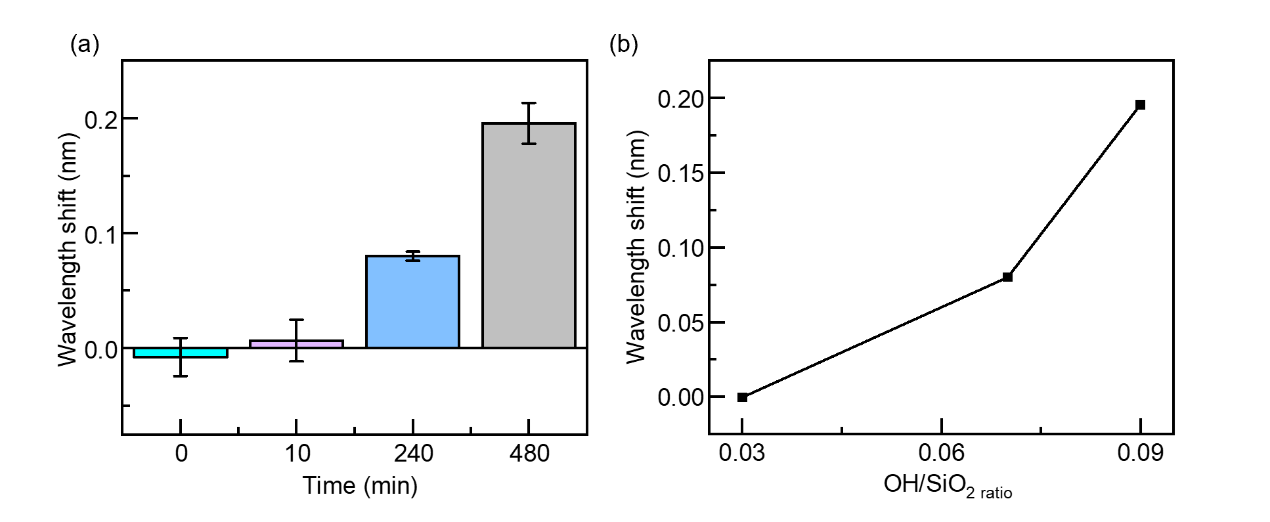


**Fig. S40**. (a) Wavelength shift of the microfiber sensor without interface responding to a 0.25 mA-current at the counter electrode. (The surface oxidant treatment time was 0, 10 min, 4 h, and 8 h. mean ± SD, n = 3). (b) Wavelength shift of the microfiber sensor without interface responding to a 0.25 mA-current at the counter electrode depending on the surface oxidation degree.
